# Are debt-for-nature swaps scalable: Which nature, how much debt, and who pays?

**DOI:** 10.1007/s13280-023-01914-4

**Published:** 2023-09-02

**Authors:** Christoph Nedopil, Mengdi Yue, Alice C. Hughes

**Affiliations:** 1 Fudan University, 220 Handan Road, Yangpu District, Shanghai, 200433 People’s Republic of China; 2 School of Biological Sciences, University of Hong Kong, Hong Kong, Hong Kong

**Keywords:** Conservation, COVID-19, Debt-for-nature swaps, Sovereign debt, Sustainable development

## Abstract

With the ongoing sovereign debt and biodiversity crises in many emerging economies, applications of debt-for-nature swaps as a dual solution for sovereign debt and nature conservation have been re-emerging. We analyze how debt-for-nature swaps (DNS) can be scaled to protect biodiversity priority areas and reduce debt burden. We build a dataset for biodiversity conservation and debt restructuring in 67 countries at risk of sovereign debt distress and show that they hold over 22% of global biodiversity priority areas, 82.96% of which are unprotected. Furthermore, we show that for 35 of the 67 countries, using conservative cost estimates, 100% of unprotected biodiversity priority areas could be protected for a fraction of debt; for the remaining countries, applying DNS would allow the protection of 11–13% of currently unprotected biodiversity priority areas. By applying interdisciplinary research combining fundamental biodiversity and economic data and methods merging, the research contributes methodologically and practically to the understanding of debt-for-nature swaps for emerging economies.

**Supplementary Information:**

The online version contains supplementary material available at 10.1007/s13280-023-01914-4.

## Introduction

COVID-19 and its aftermath has provided us with a window of opportunity to re-evaluate development models (Elliott et al. 2020; Oldekop et al. 2020): much research has analyzed how sustainable development pathways can improve livelihoods, particularly by providing aid and fiscal spending for employment under the consideration of climate, and biodiversity (Büscher et al. 2021; Stubbs et al. 2021). Much of the success of sustainable development models particularly in emerging markets, however, depends on finding a solution for the sovereign debt crisis. The sovereign debt crisis in many emerging markets in the aftermath of COVID-19 has strained the availability of resources for sustainable development and risks exacerbating the funding gap for nature (Barbier 2022), while social considerations, such as the provision of jobs and job security, have led to stimulus efforts that potentially result in economic growth without considering negative environmental consequences (Fodha and Seegmuller 2014). The debt service suspension initiative (DSSI) of the G20 and the Common Framework (World Bank 2021) provided a temporary suspension of debt service payments between May 2020 and December 2021 for up to 73 eligible countries whose debt levels became unsustainable during the COVID-19 pandemic. However, the DSSI was not extended and only delivered US$12.9 billion in relief to 48 countries, while the Common Framework had not effectively led to significant debt reorganizations as of May 2023, with only four countries having applied to be treated under this framework (Alkhareif and Moulin 2023). This highlights the challenges of such approaches as a tool for debt reduction and sustainable growth.

A pathway for reducing sovereign debt burden increasingly discussed (F4B-Initiative 2020; Simmons et al. 2021) and implemented in four instances since 2019 [e.g., in Belize (2021), Barbados (2022), Ecuador (2023)] is debt-for-nature swaps (DNS). DNS have regained prominence in the aftermath of COVID-19 (Simmons et al. 2021), particularly for emerging economies and have been discussed as a means to simultaneously ameliorate multiple sustainable development problems: reduce debt burdens, particularly in emerging economies with high external public debt, and direct funds to nature conservation or restoration, all while creating employment (Sommer et al. 2020; Dibley et al. 2021). DNS are a financial tool initially developed and applied in the 1980s to deal with this dual problem of nature loss and sovereign debt by exchanging sovereign debt for the conservation or restoration of nature. Since 2015, DNS have restructured about US$ 3 bn (Jones and Campos 2023); with singular DNS swapping debt worth up to 1.6% of national GDP.

Several recent DNS (Ecuador, Maldives) illustrate the potential of DNS in providing a solution for mobilizing funds for conservation (Jones and Campos 2023), by solving the debt crisis and national credit ratings also allowing economic recovery. However, translating DNS into action and applying it to maximize its effect on nature protection and debt reduction is complex, especially since the scalability and effectiveness of DNS are difficult to establish and their application comes with several challenges (Essers et al. 2021). DNS identification and evaluation are hampered by the factors including the complex debt negotiations between public and private debt holders, varying national interests and support of debtor countries (Simmons et al. 2021). For example, some countries may fear job loss and loss of livelihoods through expanding nature conservation, idiosyncratic natural endowments (Zhu et al. 2021); this balance of agriculture and economy versus nature increases the cost for the evaluation of “which nature” to swap debt for, and in consequence, there is an uncertainty of how much debt could be swapped for nature. The World Bank and the International Monetary Fund (IMF), two of the largest creditors in emerging economies, had accordingly evaluated an inclusion of DNS to restructure their debt in May 2021 (Shalal 2021a). While it was seen as a potentially powerful solution for overall debt sustainability (Georgieva et al. 2022), progress was stalled due to uncertainties of DNS scalability (Shalal 2021b).

To answer the question of the scalability and maximum impact of DNS for nature conservation and debt reduction, we expand on the approaches detailed by Simmons et al. (2021) by developing a more nature-based framework for identifying biodiversity priorities and include creditors beyond China. In 67 DSSI countries for which data were available (World Bank 2021), we specify concrete biodiversity priority areas of 15 biomes nestled within five major biomes (forests, grasslands, deserts, mangroves, and freshwater; to represent diversity across major non-marine systems) and analyze how much of these areas would benefit from long-term protection (i.e., “which nature”). This represents a key question on how to most effectively use debt-for-nature swaps to target conservation and overcome the lack of resources and capacity, while providing the opportunity for a long-term sustainable future for some of the worlds’ biodiversity hotspots.

### Understanding debt-for-nature swaps and benefits over other debt restructuring tools

A variety of different tools relating to debt have been developed to help finance conservation. For example, nature performance bonds or securities provide useful tools for financing conservation besides DNS (Corden 1991; Ferry 2019; Volz et al. 2020; Essers et al. 2021). However, DNS have specific advantages compared to other nature performance securities which increase the likelihood of dual debt and nature sustainability success.

**Fig. 1:**
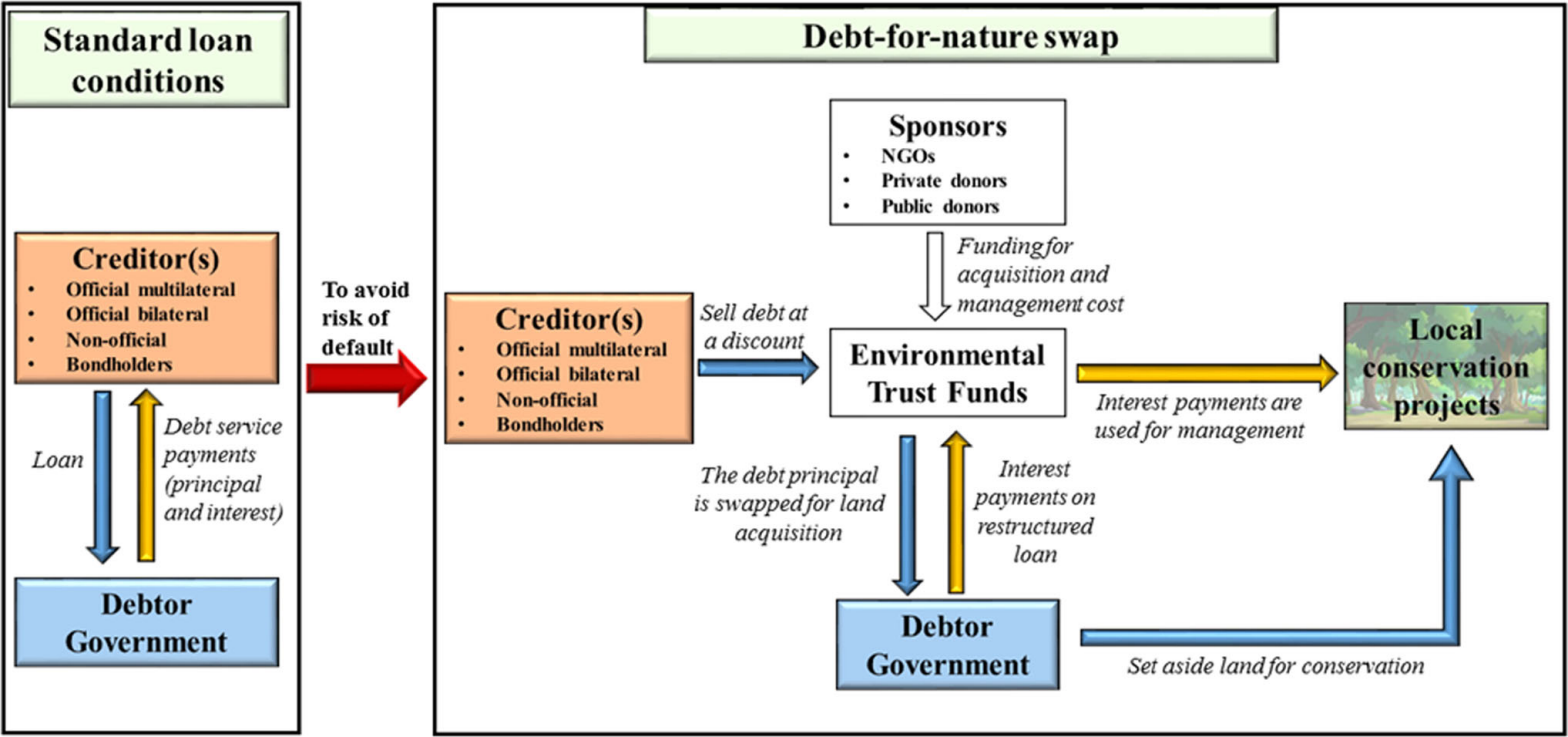
Illustration of debt-for-nature swap. Under standard loan conditions, debtor governments transfer debt service payments (including principal and interests) regularly to creditors. When the debtor government is under financial distress, a debt-for-nature swap could be initiated to avoid the risk of default while establishing and maintaining new protected areas. While multilateral creditors have so far not directly engaged in debt swaps, it would conceptually be possible

Most importantly are years of experience in designing and applying DNS to facilitate effective application, including annual funds to enable monitoring and enforcement in the Galapagos DNS (Einhorn 2023). DNS were introduced in 1984 in response to the deteriorating tropical rainforests and mounting debt obligations in developing countries (Lagos 1992; Sheikh 2018). Through a DNS, the debtor country’s debt stock is reduced in exchange for commitments of the debtor government to protect nature (UNDP 2017) (see Fig. 1 for a conceptual depiction of financial and contractual flows of DNS). The first debt-for-nature agreement was signed in 1987 between Bolivia and Conservation International (CI), in which CI purchased US$650,000 of Bolivia's foreign debt in the secondary market at a discounted price of US$100,000. In exchange, the Bolivian government set aside 3.7 million acres in three conservation areas (Shabecoff 1987). Since then, DNS have been applied in over 30 countries across all continents (Sheikh 2018). Between 1987 and 2015, the value of debt restructured under DNS agreements surpassed US$2.6 billion, resulting in about US$1.2 billion of transfers to conservation projects (UNDP 2017). The DNS in Seychelles in 2018 was worth more than 1.5% of the debtor country’s GDP and led to the protection of 30% of the Seychelles marine ecosystem (Convention on Biological Diversity (CBD) 2016). The 2021 DNS in Belize created a conservation fund worth 10% of Belize’s GDP and set aside 30% of its ocean for conservation while involving exclusively private creditors (Landers and Lee 2021). Over time, DNS have increased the amount of swapped debt from several millions per swap in the 1980s to reach US$ 1.6 billion in a single swap in 2023 (Jones and Campos 2023).

A second advantage of DNS is the continuous interest payment of the debtor government on the remaining debt to the environmental trust fund. This reduces moral hazard (i.e., the risk that the debtor country takes on undue future risk to again over-expose to debt obligations due to the previous experience of debt forgiveness), contrary to, e.g., debt forgiveness. DNS also ensure local benefits (contrary to, e.g., debt-for-resources swaps) because of domestic investments (Ferry 2019) and allow for higher permanence through the provision of PA management cost (in comparison to, e.g., nature performance bonds).

A third reason for the relevance of DNS, compared to, e.g., nature performance bonds, is that DNS are, as noted above, based on ex-ante evaluation and agreements on the nature swap and thus enable more effective conservation (F4B-Initiative 2020). In comparison nature performance bonds risk high transaction cost to provide nature protection ex-post. A fourth reason and logical consequence of the above is that DNS avoid the conflict of interest for debtor countries to choose between protecting environment for lower interest and accelerating economic development possibly at the cost of environmental protection and pay higher interest rates. Both mechanisms of DNS and nature performance securities can, nevertheless, be combined at the debt restructuring and nature protection nexus.

While studies showed the effectiveness of DNS to improve nature outcomes, such as reduction in forest loss (Sommer et al. 2020), studies also point to challenges of applying DNS (Bird 1988; Essers et al. 2021; Nedopil et al. 2023), e.g., due to requirement for strong governance in creditor countries to implement and administer a DNS, as well as long implementation time, particularly in the identification and valuation of nature.

In this study, we avoid assumptions on the effectiveness of DNS action dependent on local governance for four reasons that had been sought out as a potential “selector” for eligible countries (Essers et al. 2021; Simmons et al. 2021): first, governance in most DSSI countries is below the global average (Transparency International 2021), and finding a cut-off point for the acceptable quality of governance seems both arbitrary and would exclude too many DSSI eligible countries from DNS from the outset; second, successful cases of DNS have been implemented in countries with weaker governance, such as Belize and Colombia; third, even in countries with good governance, conservation efforts fail due to ineffective management (Watson et al. 2014); fourth, any form of debt restructuring (e.g., debt forgiveness, new debt issuance through multilateral or bilateral institutions, debt-for-resources swaps) in economies of weak governance has high implementation risks, while debt restructuring is necessary to avoid sovereign default entailing more social, environmental, and economic risks. This is also in line with Freytag and Pehnelt (2009), who found that in the early twenty-first century, debt relief was not dependent on governance quality. This brings us to the conclusion that an evaluation of DNS is relevant in all types of economies. Accordingly, we aim to provide data and a framework on where DNS could be scaled effectively to protect nature, recognizing that effective management, social inclusion, broader debt restructuring are important ingredients DNS application (Pressey et al. 2015; Essers et al. 2021; Simmons et al. 2021).

In this analysis, we address the second challenge of identifying relevant nature to conserve and thus an ex-ante understanding of applicability and scalability of DNS in specific countries (Shalal 2021b) by identifying applicable nature to swap (i.e., “which nature”), paired with the evaluation of the cost for conserving this nature (i.e., “how much nature and debt”), paired with the analysis of which creditors would need to be engaged (i.e., “whose debt”). By making the information of “which nature,” “how much debt,” and “whose debt” accessible in a rigorous framework, we aim to evaluate the potential and scalability of DNS, while also reducing information cost for potential DNS sponsors in identifying relevant nature and relevant countries.

## Materials and methods

### Identifying areas for DNS application

To identify the areas for DNS application, we focused on intact but unprotected areas, as these areas were most likely to maintain original species (important for ecosystem function), would require little if any restoration and were often under government management instead of direct private management and use, making them easier to swap for sovereign debt. Furthermore, the need to stem continued loss of intact and high diverse ecosystems will be paramount to achieving future conservation targets (Maxwell et al. 2020). The focus on intact land also reduces the risk of displacing economic activity, local livelihoods, or other land rights, reducing the economic impacts as well as the costs. Rather, as many countries struggle to recover economically from the COVID-19 pandemic, alternate jobs and livelihood provision is crucial for economic recovery, an opportunity that could be offered by investing in protection and developing ecotourism. Finally, this choice also provides a more cost-effective solution than restoring already-degraded lands, for which local governments might not have sufficient resources to pay for, even with a debt swap.

We focused largely on terrestrial ecosystems as they face direct threats due to damage and loss instead of from displaced activities such as chemical or climate-related issues (prevalent, e.g., in marine systems). Terrestrial ecosystem problems [deforestation (Sommer et al. 2020), over-grazing (Smith 2017), rapid loss of mangroves (Thomas et al. 2017; Goldberg et al. 2020; Richards et al. 2020), and river fragmentation (Grill et al. 2015)] may be targeted most effectively with local spatial conservation approaches. Clearly, marine systems are also threatened, and managing fishing sustainably, reducing pollution, and managing climate change as part of a complex suite of mitigation strategies may be effective at reducing marine diversity losses, especially as protection may be undermined by activities outside the protected area in marine systems (Agardy et al. 2011).

However, the pressure on terrestrial ecosystems may increase further, given the post-pandemic stimulus of economic growth through infrastructure development, underscoring the urgency of effective protection before these areas are modified. Identifying and protecting such areas is also crucial, for example, to avoid the construction of infrastructure near naturally protected areas through initiatives such as China’s Belt and Road Initiative (Hughes et al., 2019). Intact habitats must be a priority for protection as the recovery of ecosystems can be slow, and restoring ecosystem function is challenging to assess, especially if certain keystone species are not present. Based on past trends, many developing countries have the highest rates of population growth and habitat loss, and thus proactive means to prevent the loss of intact systems reduce conflicts of interest with existing owners, maximize the potential retention of biodiversity, and reduce the costs of restoring ecosystem functionality.

Determining priority areas for conservation presents a challenge, as there are many alternatives on how they can be identified, and selecting appropriate indices is a continued topic of debate (Kukkala and Moilanen 2013) (for a full overview of our methodology on nature, including the datasets used, see the Supplements). All approaches have inherent compromises, yet developing metrics to identify potential biodiversity hotspots in the absence of accurate data for many regions means that measures should identify the potential hotspots without the risk of confounding issues due to data quality. Here, we aimed to identify areas that still retain diversity (which is most efficient both on cost, and species retention, as well as not contravening local access rights), but due to rapid rates of deforestation, grazing, and other forms of degradation, are without intervention likely to be lost (and converted into economically productive land). Rates of habitat loss remain high across many global biodiversity hotspots and ensuring rates of loss are stemmed is more efficient than considering restoration of areas that have already lost much of their biodiversity.

Species richness indices are frequently used to provide a metric of biological diversity (Jetz et al. 2012; Jenkins et al. 2015; Di Marco et al. 2019; Jung et al. 2021), despite many of these patterns resulting primarily from common species (Jetz 2002). Yet the quality of available global biodiversity data records of species occurrence are spatially or taxonomically biased (Hughes et al. 2021a, b). This makes comparisons between regions or taxa challenging or impossible (Hughes et al. 2021a). Identifying areas for priorities needs to overcome spatial biases in commonly used datasets, to ensure hotspots are captured, including areas with insufficient data to assess their species composition.

To circumvent the challenges of identifying key areas in the face of these data biases and gaps, we identified areas with low/no disturbance (based on an updated version of the human footprint map (Venter et al. 2016), as rapid rates of change in many of these regions necessitate updating all components of the original footprint). We then identified the most productive parts of 15 biomes based on productivity data. Productivity was used as a surrogate indicator for richness as it can be sensed remotely and overcomes the data shortfalls. To ensure it is a reliable indicator, we cross-referenced it with key biodiversity areas and richness based on IUCN and Birdlife to ensure these hotspots do provide key areas for biodiversity (see supplements for full details). Biomes were used to ensure representative protection across taxa and biomes (Maxwell et al. 2020). Using productivity as a surrogate of available energy for the ecosystem, and therefore diversity, removes spatial errors due to uneven data collection and digitisation efforts (Hughes et al. 2021a; Hughes et al. 2021a,b). By identifying the most productive parts of each intact biome, we highlight the areas with the greatest ability to support biological diversity, as productivity has been shown to be a correlate of species richness in many studies (Hobi et al. 2017; Coops et al. 2018; Silveira et al. 2021).

To assess the appropriate productivity metric, we compared the overlap with hotspots identified using IUCN and birdlife data for several different productivity metrics, and explored the overlap with Key Biodiversity areas. This comparison assesses if hotspots are retained to ensure known hotspots are captured, but areas missed by these datasets (i.e., due to political borders) are encompassed by using an index that is standardized and representative across the planet, as it does not require human decisions compared to species-based indices. Once the most potentially diverse parts and the percentage of priority areas falling into each country have been identified, the relevant percentages of five major biomes (forest, grassland/savanna, desert, riparian and freshwater, and mangrove) were calculated. To ensure our targets consistently captured known sites of diversity, we compared our results to existing maps of richness for four vertebrate and one invertebrate taxa, while avoiding potential biases inherent in many of these maps. We thus aimed to show a high degree of overlap with areas identified in those datasets, and to circumvent issues associated with political boundaries and other biases that may underestimate diversity in some regions based on such data. These potential richness maps were also used to determine which productivity was the best potential surrogate for species richness. Finally, we analyzed how much of those priority areas are currently unprotected using the database of global protected areas. This analysis was first conducted at a global level, then for the 67 DSSI countries for detailed analysis, further details and all percentages are given in discussion.

### Cost evaluation

Estimating the amount of debt to be swapped requires specifying the cost to establish and manage protected areas. This, however, is challenging: surveys on the acquisition and operating costs for protected areas, as well as their ratios, vary both across and within regions and biomes by factors of 50 (Waldron et al. 2020). To nevertheless estimate the cost (for a detailed overview of the cost used for the estimation, see Supplemental text and Tables S2 and S3), we analyzed the data from previous and current DNS (e.g., Seychelles) and data on conservation cost (Waldron et al. 2020) including more granular, albeit older, conservation cost data provided by James et al. (2001). Throughout our calculations, we adjusted the cost data based on James et al., to 2020 US$ and provided sensitivity analyses using a range of plus and minus 15%. We estimated the annual operating cost at 31% of acquisition cost (Bohorquez et al. 2019) on average, recognizing high variability depending on, e.g., the frequency of patrols, type of patrol, the area size, and local salaries. Similar to acquisition cost, we used conservative estimates for operating costs.

### Creditor engagement evaluation

Estimating the value of nature in relation to the debt to evaluate scalability requires a detailed analysis of “public external debt stock” as the type of debt relevant for analysis available on the World Bank website for each eligible country. In this term, “public” refers to debt owed by a public agency or a private agency with a public guarantee in the debtor country, and “external” refers to debt owed to nonresidents (World Bank 2020). By this, we made sure that our analysis aligned with the cases of past debt-for-nature swaps, which were generally implemented between the debtor government and one or more foreign lenders (usually the creditor governments) or bondholders (e.g., Belize, Ecuador). The latter type of DNS require debtor countries to have access to capital markets, a willingness of a usually foreign entity to guarantee the newly issued debt (as was the case in Belize through the US International Development Finance Corporation), as well as the availability of an underwriter (e.g., in the Belize case, Credit Suisse was underwriting a blue bond) (Nedopil et al. 2023).

We narrowed down the group of debtor countries under analysis to the 73 countries eligible for debt service suspension initiative (DSSI) as they are more vulnerable to debt distress and have a higher priority for debt restructuring, including debt swaps. In response to COVID-19, the G20 established DSSI in May 2020 to provide temporary suspension of debt service payments owed to official bilateral creditors until December 2021 (World Bank 2020).

Debt data for 67 out of the 73 DSSI eligible countries (detailed data for South Sudan, Micronesia, Tuvalu, Kiribati, and the Marshall Islands are not available and Kosovo is not universally recognized as a country) were obtained from the World Bank International Debt Statistics (IDS). IDS contains country-level data of public external debt stocks owed to four types of non-resident lenders: (1) official multilateral creditors, i.e., international organizations whose membership and decision-making process includes the government of two or more countries, such as the IMF, the World Bank, Asian Development Bank; (2) official bilateral creditors, i.e., lending by sovereign governments and all public institutions in which the government share is 50 percent or above and encompassed of the general government, central government; state and local government; central bank; and public enterprise; (3) bondholders, i.e., holdings of securities by investors for which the issuer has promised to pay a specified amount of money at a fixed date and income at periodic dates until maturity, including publicly placed bonds and privately placed bonds; and (4) non-official creditors including all other private creditors, such as those that are officially supported by an export credit guarantee, or other forms of risk-mitigating guarantee, from an official bilateral entity or multilateral institution (World Bank 2020).

The sizes of total and disaggregated public external debts were measured as percentages of the debtor country’s GDP (debt-to-GDP ratios), in line with the IMF Debt Sustainability Analysis (IMF, 2020). GDP data for individual debtor countries were obtained from the World Bank, except Somalia’s from IMF. We used 2019 data as they were the most current and complete data available, while assuming that the overall debt levels and debt ratios in the 67 countries would be higher in 2020 due to economic recession and increased public spending.

## Results and discussion

### Scaling debt-for-nature swaps

**Fig. 2:**
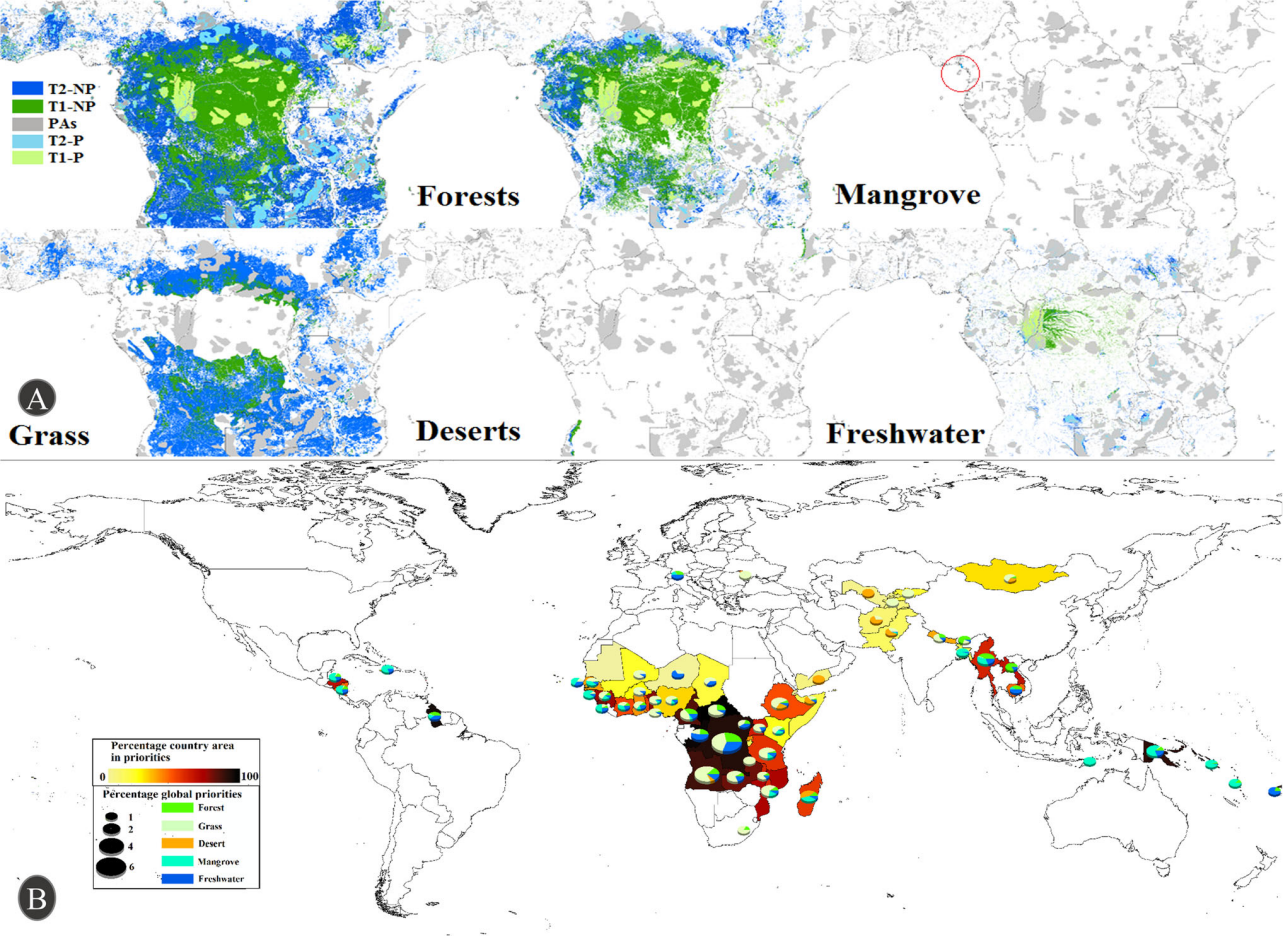
Priorities for conservation. **A** High-resolution priorities for the different biomes across Central Africa. Note that some habitats are mixed, so may include priorities under more than one “biome” type. Top priority (T1) areas are shown in green, whereas second-level priorities (T2) are shown in blue. Protected priority areas are shown as paler and gray areas are protected areas that do not cover priority areas. **B** Global priorities for biodiversity within the 67 DSSI countries. Ramp (yellow-black) shows the percentage of global priority overall, pie-chart size indicates the priority as a proportion of global priority areas (so larger pies indicate larger proportions of all global priorities, and the fractions of each color indicate the percentage from each biome)

Overall, the 67 DSSI countries host 22.31% of global biodiversity priority areas, while only 17.03% of these priority areas are protected (Table 1; Fig. 2A). Notably, only 47.7% of protected areas (PA) overlapped with priority areas in these countries, highlighting the importance of protected area placement: Somalia, Liberia, and Djibouti have some of the lowest percentages of national priority areas protected at 0–0.05%. Even as the area protected increases, protection of priority areas may not if suboptimal land is protected (e.g., Haiti protects 2.17% of its land, but only 0.28% of its priority). Conversely, some countries with little overall protection protect most remaining priorities (e.g., Bangladesh protects 4.43% of land area and 76.65% of its priority areas). This highlights the need for targeted intervention, and that the majority of key areas lack protection at present.

**Table 1:** Overview of priorities for biodiversity

	Total share of global priority areas in selected 67 countries (%)	Percentage of priority areas protected within the 67 countries (%)	Percentage of priority areas protected globally (%)	Top five countries with the highest priority	Highest priority country’s share of global priority (%)	Protected priority-area for biome in highest priority country (%)
Total	22.32	17.03	15.14	1. DR Congo 2. Angola 3. Mozambique 4. Central African Rep 5. Papua New Guinea	5.20	12.70
Forest	19.71	16.93	18.66	1. DR Congo 2. Angola 3. Central African Rep 4. Papua New Guinea 5. Cameroon	6.47	14.81
Grassland	50.63	16.61	13.75	1. Angola 2. DR Congo 3.Central African Rep^a^ 4. Mozambique 5. Ethiopia	9.51	4.71
Desert	5.34	19.63	10.25	1. Madagascar 2. Ethiopia 3. Angola 4. Mongolia 5. Pakistan	1.91	21.91
Mangrove	20.26	30.15	34.46	1. Papua New Guinea 2. Solomon Islands^b^ 3. Madagascar 4. Mozambique 5. Myanmar	7.41	4.41
Freshwater	21.57	28.13	22.92	1. DR Congo 2. Papua New Guinea^c^ 3. Angola 4. Zambia 5. Guyana	7.01	4.08

^a^For grassland Zambia would be in 3rd position but as 40.01% of the priority-area is protected it has not been listed here

^b^For mangrove Bangladesh would be in the second position, but as 99% of the priority for mangroves are already protected this would not be useful

^c^For freshwater the Republic of Congo would be second, but with 74.8% of the priority protected we have not listed it here

Areas with under 1% of global priority for that biome have been italicized

### Biome-specific debt-for-nature swaps

To allow for biome-specific DNS (e.g., in the interest of specific DNS sponsors), we identified priority areas and assessed their protection across biomes (see Table 1). Assessing priorities on a per-biome basis maximizes ecological representativeness, which may otherwise miss regions with lower absolute diversity but unique species assemblages. We assessed the percentage of global priorities that fell into each country for each biome, as well as explored what percentage of land, and priorities were covered by priorities or protected at a national level.

For forest priorities (based on current forest coverage), DR Congo hosts 6.47% of the Earth’s priority forest areas (Fig. 2A, B). These areas make up 62% of DR Congo’s land area, yet protected priority areas only make up 9% of DR Congo’s area covering 15% of its forest priorities (Fig. 2). A drastic case is Papua New Guinea, which hosts 1.8% of global top priority forest areas, equating to 60% of the island's land area. Yet protected priority areas only make up 1.2% of the island, and 2% of national priority areas. With high rates of development in these regions, and the potential for high development especially in Papua New Guinea and the DR Congo, targeted interventions with sound financial elements may be crucial to maintaining biodiversity in these regions. Rates of forest loss, development and exploitation for oil in these regions (DiGirolamo 2022; Graham 2022) highlight that if efforts are not made to change the increased trajectory of loss irrecoverable changes (and associated extinctions) will occur). Attention on these issues, such as recent engagement with CIFOR and ICRAF (Koffi 2023) could provide the opportunity to discuss approaches like the implementation of DNS as a means to conserve diversity and secure livelihoods in this region. Similarly in Papua New Guinea the illegal production of timber poses the greatest threat to the region (DiGirolamo 2022). Furthermore as well as hosting crucial areas for biodiversity these areas would be prime areas for DNS to be applied.

For grassland priorities, Angola hosts 9.5% of global priority areas. These priority areas occupy 78.92% of the country’s land area, of which only 4.72% are protected. Similarly, DR Congo is a major priority for grasslands, with 9.47% of the Earth’s priority grassland, of which only 9.51% are protected. For desert priorities, few DSSI countries host desert priority areas: among those, Madagascar hosts 1.9% of global desert priority areas, of which 21.91% are protected. These ecosystems are often overlooked, though hunting and over-grazing present major risks to grassland habitats, as does the planting of trees under the guise of “Nature based solutions” despite trees not really belonging in these ecosystems (Orr and Hughes 2022). The diversity of these regions can be particularly sensitive and deserves heightened attention to avoid loosing frequently neglected diversity in ecofragile regions (Zhang et al. 2023).

For mangroves, Papua New Guinea hosts 7.41% of global priority areas. These equate to less than 0.4% of Papua New Guinea’s land area. Nevertheless, only 4.42% of these mangrove priority areas are protected. Bangladesh hosts the next greatest mangrove area, (2.32% of global priority areas), representing 0.4% of the country. Bangladesh protects almost all (98.96%) of its mangroves and would thus not necessarily represent a good choice for mangrove-specific DNS. The Solomon Islands host 1.64% of global priority areas, which make up less than 1.4% of the land area, of which only 5.65% are protected. Like many other native systems, Mangroves are also in decline (Bhowmik et al. 2022; UNEP 2023), and while mangroves are growing in new areas due to upstream deforestation, without renewed attention diverse mangrove systems may be lost across much of their native range. For freshwater priorities, DR Congo hosts 7.02% of global priority areas, making up 9.34% of its landmass of which 17.94% are protected. This is followed by Papua New Guinea which is home to 2.2% of global freshwater priority areas, of which 4.08% are protected. While protected areas provide little protection from upstream pollution or damming (both of which present major threats to freshwater systems), they can at least protect riparian areas and reduce the pressure of over-harvest within protected systems (Azevedo‐Santos et al., 2019). Furthermore, globally only around 15% of inland water is protected, and this has actually decreased over the last 30 years in many countries (Bastin et al. 2019). With the target of 30% for all systems under the Kunming-Montreal Global Biodiversity framework, identifying key regions to meet the target is crucial (Hughes 2023).

### Country-specific debt-for-nature swaps

For country-specific DNS, e.g., to maximize priority-area protection within Aichi target 11 of 17% PA coverage or 30% as the Kunming-Montreal framework (Convention on Biological Diversity (CBD) 2022; Hughes 2023), knowledge of priority areas relevant for effective protection in each country is required.

We calculated the percentages of unprotected priority areas for each country and biome, analyzing relevant coverage we identified specific national targets for DNS to maximize national and global priority-area protection (Fig. 2B). DR Congo has the highest priority areas, which hosted 5.2% of global surface area across all priority areas, occupying 88% of DR Congo’s land area, of which 12.69% are protected. In African countries, DNS have significant potential for grasslands. Tropical countries, like the Solomon Islands, Papua New Guinea, and Madagascar, are most in need of mangrove protection, which additionally provides key services from insulation from Tsunamis to maintaining a healthy marine ecosystem (Hughes 2017). Furthermore, countries like DR Congo have seen rapid rates of deforestation, having lost 8% forest coverage since 2000, and around 0.7% annually for the last four years (Global Forest Watch) Data S1).

### How much debt for nature?

The average PA acquisition cost per km^2^ ranges from US$1,535 in sub-Saharan Africa, to US$17 350 in the Pacific. Using the spatial data evaluated above, we found that the total acquisition of unprotected areas cost for the 67 countries would amount to US$26.6 billion, which would equal 5.06% of these countries’ total sovereign debt. This would, overall make the application of DNS scalable and relevant, especially if further funds are provided from the debt to contribute to management costs annually (as the Galapagos DNS have been structured, Einhorn 2023).

**Fig. 3:**
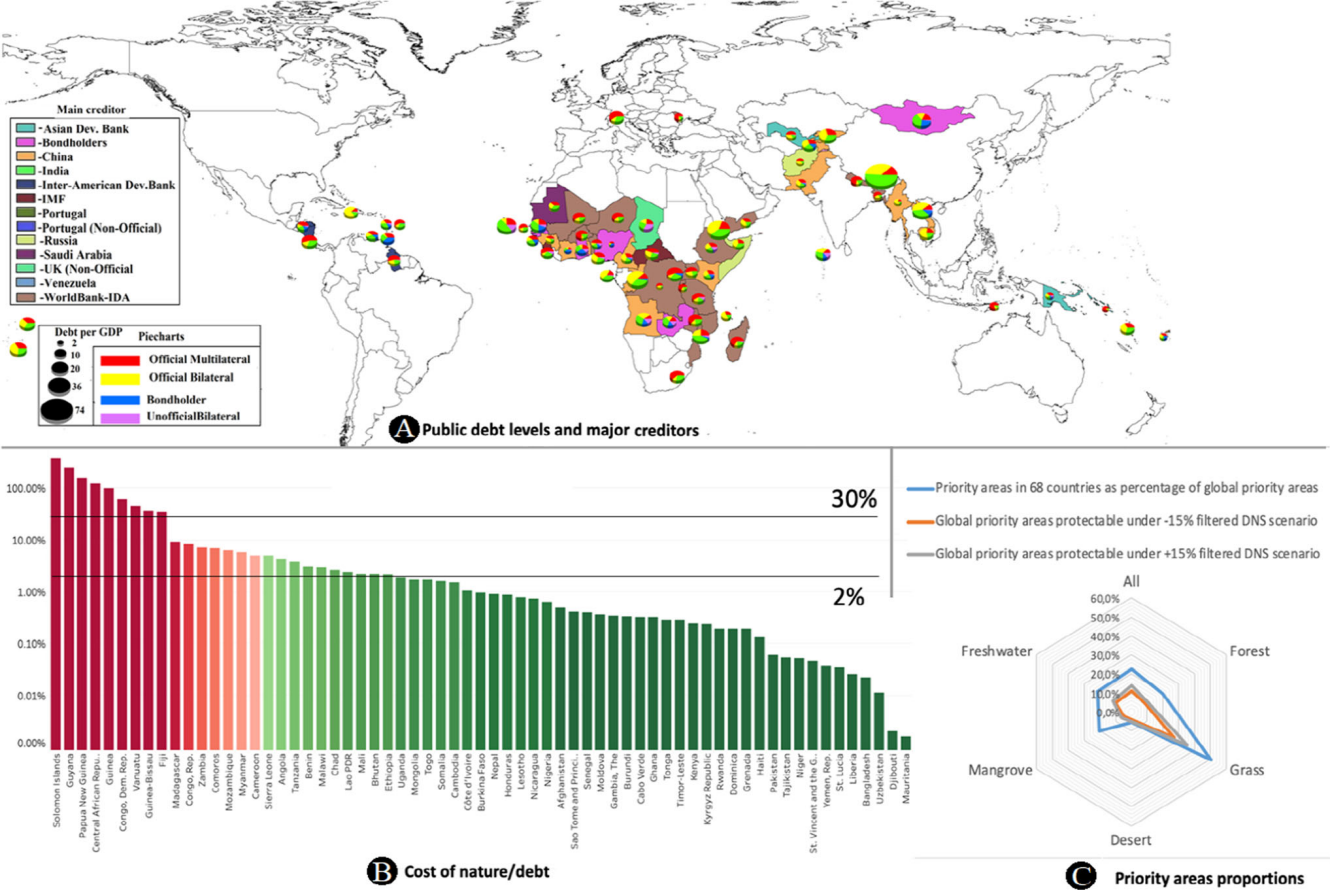
Cost of protection. **A** Public external debt and largest creditor in 67 DSSI eligible countries in 2019. The sizes of pie charts refer to the overall public external debt-to-GDP ratios (ranging from 2 to 74%), proportions of debt types are shown in pies, highlighting the degree of debt for each country and the type. The colors of the main creditors are shown as country colors, notably many African countries are largely credited by the World Bank, followed by China. **B** Cost for priority-area acquisition as % of public debt with 2% and 30% thresholds. **C** The proportion of global priority-area protection under different scenarios: e.g., the 67 countries host 50.6% of global grass priority areas. With the edge scenarios of −/+ 15% cost compared to the baseline cost, 27.31%/35.36% of the Earth’s grassland priority areas could be conserved with the 2% minimum and 30% maximum level of debt swap. In the scenario with + 15% higher cost for PA acquisition, more countries would pass the 2% minimum threshold, making the protected area larger

Yet, due to idiosyncratic country endowments, the scalability of DNS varies across countries; for 35 countries, the value of all priority areas is less than 0.1% of public debt, making those countries less relevant for DNS, though still feasible as annual installments to run DNS would require a greater amount than soley land purchase, for four countries even 100% of their public debt would not suffice to acquire unprotected priority areas (Solomon Islands, Papua New Guinea, Guyana, and the Central African Republic—Fig. 3B), requiring more decisions on which nature to swap, or a more complex structure to factor management costs. These results changed slightly when applying sensitivity checks with the + 15%/-15% cost change: in 32/36 countries, the acquisition cost would amount to 0.1% and less, while similarly, 100% of public debt would not suffice to acquire currently unprotected priority areas for 5/4 countries. Across the 67 countries, the median acquisition cost for nature is 0.91% of public debt (-15% sensitivity: 0.77% + 15% sensitivity: 1.04%) to acquire all priority areas. In absolute amounts, this equals US$ 54.5 million (-15% sensitivity: US$ 47.6 million, + 15% sensitivity 64.4 million). The median cost is well below the value of previous debt swaps (e.g., the nature conservation value of the 2023 Ecuador DNS is estimated at US$323 million as part of the USD1.6 billion debt buyback).

This means that for about half of DSSI countries, a small fraction of the outstanding debt could be swapped and would suffice to protect currently unprotected priority areas. If used in combination with annual installments for running newly protected areas this approach provides an effective opportunity for protection, and would bridge the major funding gap in conservation as highlighted in the Target D of the Kunming-Montreal Global biodiversity framework (Hughes 2023).

To evaluate the DNS applications in those countries where DNS could be scaled, we filter countries whose unprotected nature is worth a minimum of 2% of their outstanding debt, while we limit the amount of debt available to swap for nature at 30% of the value of the outstanding debt (e.g., the Belize DNS were worth 32.5% of the original debt). This would allow 26 countries (− 15% sensitivity: 23; + 15% sensitivity: 27) to apply DNS effectively and would result in debt swaps worth US$11.0 billion (− 15% sensitivity: US$9.1 billion, + 15% sensitivity: US$12.5 billion). The sum of these DNS would result in the protection of 13.49% (− 15% sensitivity: 11.03% + 15% sensitivity: 13.94%) of the Earth’s currently unprotected priority areas, in particular, grassland, forest, and freshwater biomes (see Fig. 3C).

Besides acquisition costs, PA management costs need to be covered and provide a means to further structure the DNS to include annual sums for management to help guarantee the longer-term success of the DNS, or building in training of local staff to enable both protection and the potential for ecotourism development. Part of these can be paid for through “re-routed” interest payments on the original debt. Based on previous DNS, we conservatively assumed a 30% discount on the swapped debt (e.g., the 2018 Seychelles DNS had a discount of only 5.4%) and a 3% interest rate on outstanding debt. Across the 67 countries, this could raise US$454 million (− 15% sensitivity: US$407 million, + 15% sensitivity: US$493 million) annually, and US$232 million (− 15% sensitivity: US$193 million, + 15% sensitivity: US$262 million) in the filtered scenario. These interest payments would only cover a maximum of about 6.8% of the assumed management cost. The annual management cost gap would amount to US$ 6.2 billion (− 15% sensitivity: US$5.5 billion, + 15% sensitivity: US$ 6.7 billion) across all countries, and US$3.2 billion (− 15% sensitivity US$2.6 billion, + 15% sensitivity US$3.6 billion) in the filtered scenario. These costs would need to be covered through other domestic or international sources, which requires further negotiation and fundraising. Possibly, part of the annual US$100 billion global climate finance commitments to developing countries reiterated during COP26 could be utilized, where biodiversity protection has been recognized as an element of climate mitigation and adaptation. It should also be noted that climate finance has in some cases been misused and actively increased deforestation great care is needed to monitor implementation as well as aligning climate and biodiversity goals (The Gecko Project 2023). In addition, there is major opportunities to align climate and biodiversity goals to better serve both (Zhu et al., 2021).

### Whose debt?

The 67 countries had about US$522 billion public external debt stocks at the end of 2019. Public external debt as a percentage of GDP varies greatly across the 67 countries: in 10 countries, the ratios are higher than 50%, including Bhutan (104%), Cabo Verde (92%), Mozambique (73%), Djibouti (65%), Mongolia (60%), Sao Tome and Principe (58%), Lao PDR (57%), Mauritania (56%), Senegal (55%), and Zambia (51%). For most (55/67) countries, the ratio falls between 10 and 50%. 46% of debt is held by official multilateral creditors (e.g., World Bank), 34% by official bilateral creditors (e.g., China), 13% by bondholders, and 7% by non-official creditors (Fig. 3A). Some or all of this debt could be used for DNS.

Depending on the DNS scenario and depending on whether multilateral institutions such as the IMF and World Bank participate in DNS (which currently is not possible), different creditors could swap different amounts of debt to achieve the protection of the identified unprotected areas. For each of the filtered scenarios, we calculated the values of debt for each bilateral, multilateral and private creditor using two assumptions: with and without the participation of multilateral financial institutions. We continued to apply the sensitivity analysis of ± 15% cost.

**Fig. 4:**
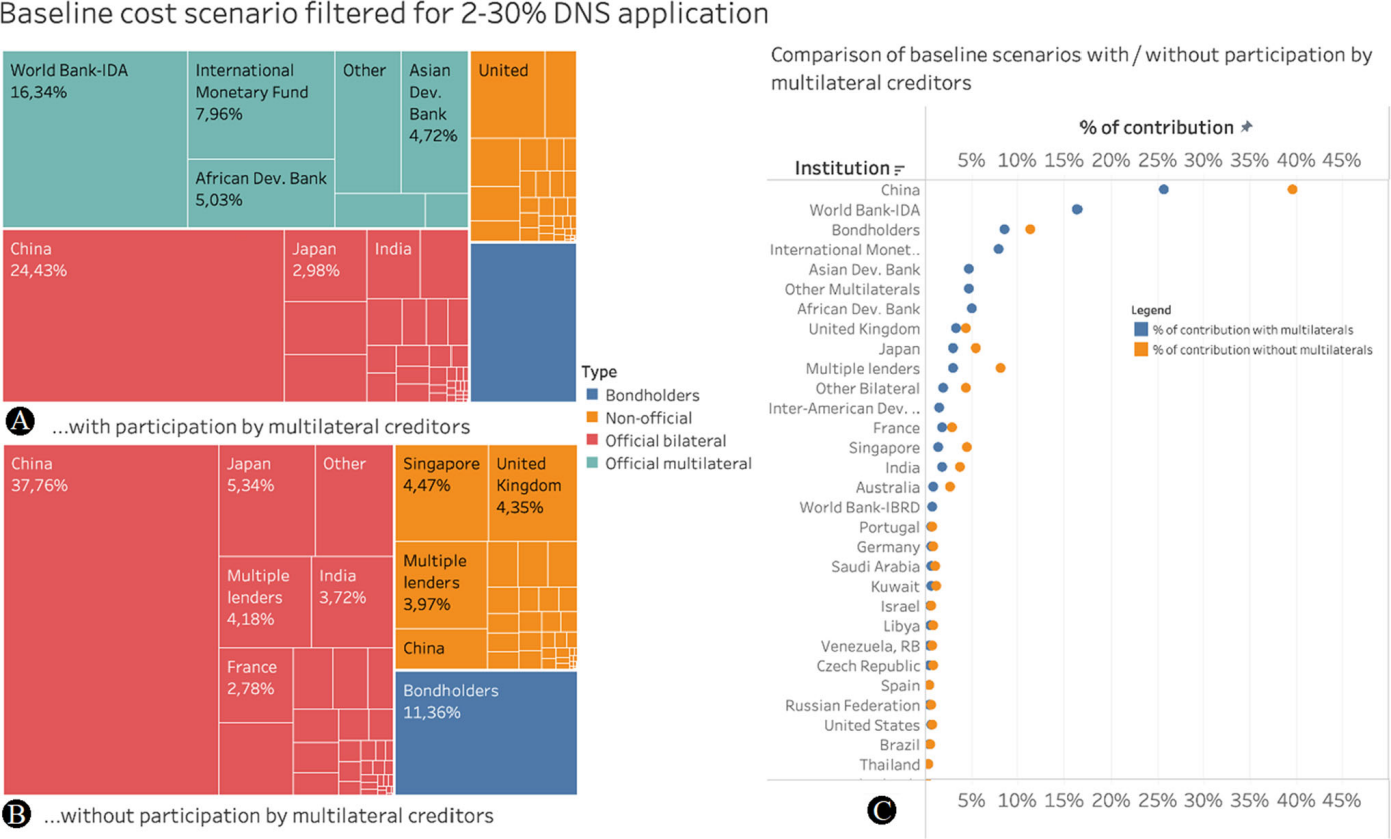
Cost of debt-for-nature swaps for creditors (at baseline scenario with minimum 2% and 30% debt-for-nature swap. **A** with participation by multilateral creditors, **B** without the participation of multilateral creditors, and **C** cost per creditor in comparison in the two scenarios, showing China’s contribution rising from 24.43 to 37.76%

Assuming equal participation in the DNS of all creditors including multilateral institutions, multilateral and official bilateral creditors would have to provide most of the debt for swapping: The World Bank would have to provide US$ 1.84 billion (− 15% sensitivity: US$1.40 billion, + 15% sensitivity: US$2.15 billion), while China (through both official and non-official bilateral debt) would have to provide US$2.77 billion (− 15% sensitivity: US$2.29 billion, + 15% sensitivity: US$3.16 billion). Private bondholders would be the third-largest group to support the envisaged DNS (US$1.04/0.93/0.8 billion) (see Fig. 4A) and would provide a viable and simple to structure mode for DNS application.

Under the assumption that multilateral lenders would not participate in a DNS, the distribution of debt to be swapped shifts toward bilateral lenders (both official and unofficial) and bondholders. In the baseline scenario, China would have to shoulder 39.6% of the debt to be swapped, worth US$5.5 billion, followed by bondholders (11.4%, or US$1.6 billion) and the next largest bilateral lender being Japan (5.41%, or US$0.75 billion) (see Fig. 4B—a full analysis of creditor countries involvement in different scenarios is in Table S4). Given China’s role in international development, working to build conservation alongside this development provides a real opportunity for sustainable conservation finance.

### Applying new tools

The aftermath of the COVID-19 crisis has provided a unique opportunity to consider applying DNS to conserve key priorities in many emerging countries, with National debt high, but new opportunities for ecotourism development with the return of travel. DNS allow debtor countries in debt distress to reduce their debt burdens, while simultaneously establishing protected areas. While previous literature has raised the awareness of debt-for-nature swaps for sustainable development (Essers et al. 2021; Lazard 2021; Simmons et al. 2021; Volz et al. 2020), this article expands this literature by highlighting potentials and limitations of debt-for-nature swaps as an easily scalable tool to reduce sovereign debt burdens (Essers et al. 2021).

We achieved this evaluation of scalability by applying a new model and using new data to analyze the questions of “which nature, how much, and who pays” for DNS across 67 of the countries under the debt service suspension initiative (DSSI) for which consistent data were available. We found that for about half of the 67 countries, protection of all currently unprotected areas would be possible, but taking lessons from recent DNS to provide ongoing costs to implement the DNS would also need to be considered for the long-term success. We found that DNS have the potential to swap US$12 to 15 billion for key areas alone, which would allow the conversion of 11–13% of the earth’s current unprotected priority areas into protected areas, in particular grasslands, freshwater, and deserts. Should land acquisition costs be higher, more countries could fall within the threshold of scalability, but also risk setting higher cost standards for future application of DNS. Alternatively, scalability could conceptually be improved by including not only currently unprotected priority areas, but also restoration. However, with a higher implementation cost for restoration than for conservation, paired with restricted availability of public finance in these countries, this solution seems to be more difficult to implement.

As management cost of the PAs is about US$3.4 billion and higher than expected interest payments, 93% of these cost would have to be covered through newly raised funds, or additional measures within the DNS (such as annual installments, as in the case of the recent Galapagos DNS, Einhorn 2023). Through an increasing political will to jointly tackle climate and biodiversity risks, as witnessed for example by including biodiversity commitments at COP26 in Glasgow and the commitments to conserve 30% of nature at CDB COP15, we see an opportunity to utilize parts of the annual US$100 billion climate finance commitment from developed to developing countries. Furthermore, these considerations are touched upon within Goal D of the Kunming-Montreal Global Biodiversity Framework (Hughes 2023).

## Conclusion

We find opportunities and limitations for the efficient and effective application of DNS in the DSSI countries. As a means to alleviate debt while providing nature protection, it is particularly applicable to about half of DSSI eligible countries and would greatly facilitate the effective conservation efforts in key regions in these countries. This tool also overcomes the major challenge of biodiversity finance highlighted within the COP15 discussions, as conservation targets can only be achieved if the finance exists, that means to bridge the finance gap such as DNS provides a key tool to enabling countries to meet current and future conservation targets. The potential to apply DNS as an effective conservation tool is inarguable, yet maximizing gains requires a clearer understanding of costs, local capacity, and political willingness with creditors and debtors. Furthermore, modes of engagement to ensure metrics are meaningful indicators of biodiversity conservation are required, while effective engagement to increase the social and economic benefits, as well as those to biodiversity, must be ensured. Frameworks such as that outlined here cross-reference that information to guide the efficient application of DNS and identify the key regions to maximize benefit. By focusing on countries identified in this paper, a faster application of DNS could be achieved, providing relevant lessons as an effective way to enable countries to meet commitments and targets nationally, and for global conventions such as the CBD.

### Supplementary Information

Below is the link to the electronic supplementary material.

Supplementary file1 (PDF 806 kb)

Supplementary file2 (XLSX 36 kb)

Supplementary file3 (XLSX 78 kb)

Supplementary file4 (XLSX 16 kb)

Supplementary file5 (XLSX 568 kb)

Supplementary file6 (XLSX 14 kb)

Supplementary file7 (XLSX 568 kb)

## Data Availability

All data used for analysis are cited in text as appropriate and listed in Table S1 or collated within tables as part of supplementary data. Full tabular results for nature are available as Data S1.

## References

[CR1] Agardy T, di Sciara GN, Christie P (2011). Mind the gap: addressing the shortcomings of marine protected areas through large scale marine spatial planning. Marine Policy.

[CR3] Alkhareif, R.M., and E. Moulin. 2023, April 19. *What the Chad debt deal means*. FinDevLab. https://findevlab.org/news_and_event/what-the-chad-debt-deal-means/

[CR2] Azevedo-Santos VM, Frederico RG, Fagundes CK, Pompeu PS, Pelicice FM, Padial AA (2019). Protected areas: a focus on Brazilian freshwater biodiversity. Diversity and Distributions.

[CR4] Barbier EB (2022). The policy implications of the Dasgupta review: land use change and biodiversity. Environmental and Resource Economics.

[CR5] Bastin L, Gorelick N, Saura S, Bertzky B, Dubois G, Fortin MJ, Pekel JF (2019). Inland surface waters in protected areas globally: current coverage and 30-year trends. PLoS ONE.

[CR6] Bhowmik AK, Padmanaban R, Cabral P, Romeiras MM (2022). Global mangrove deforestation and its interacting social-ecological drivers: a systematic review and synthesis. Sustainability.

[CR7] Bird G (1988). Debt swapping in developing countries: a preliminary investigation. The Journal of Development Studies.

[CR8] Bohorquez JJ, Dvarskas A, Pikitch EK (2019). Filling the data gap—a pressing need for advancing MPA sustainable finance. Frontiers in Marine Science.

[CR9] Büscher B, Feola G, Fischer A, Fletcher R, Gerber J-F, Harcourt W, Koster M, Schneider M, Scholtens J, Spierenburg M, Walstra V, Wiskerke H (2021). Planning for a world beyond COVID-19: five pillars for post-neoliberal development. World Development.

[CR10] Convention on Biological Diversity (CBD). 2016. *Seychelles fact sheet*. Convention on Biological Diversity (CBD). https://www.cbd.int/doc/meetings/mar/soiom-2016-01/other/soiom-2016-01-seychelles-01-en.pdf

[CR11] Convention on Biological Diversity (CBD). 2022, December 19. *COP15: nations adopt four goals, 23 targets for 2030 in landmark UN biodiversity agreement*. Convention on Biological Diversity. https://www.cbd.int/article/cop15-cbd-press-release-final-19dec2022

[CR12] Coops NC, Kearney SP, Bolton DK, Radeloff VC (2018). Remotely-sensed productivity clusters capture global biodiversity patterns. Scientific Reports.

[CR13] Corden WM (1991). The theory of debt relief: sorting out some issues. The Journal of Development Studies.

[CR14] *Debt for nature swaps*. n.d. UNDP. https://www.sdfinance.undp.org/content/sdfinance/en/home/solutions/debt-for-nature-swaps.html#mst-1. Accessed 29 Oct 2020

[CR15] Di Marco M, Ferrier S, Harwood TD, Hoskins AJ, Watson JEM (2019). Wilderness areas halve the extinction risk of terrestrial biodiversity. Nature.

[CR17] Dibley A, Wetzer T, Hepburn C (2021). National COVID debts: climate change imperils countries’ ability to repay. Nature.

[CR16] DiGirolamo, M. 2022. ‘Carbon cowboys’ and illegal logging. Mongabay.

[CR300] Einhorn, C. 2023. Ecuador Strikes a Landmark Deal to Protect the Galápagos, and Save Cash. The New York Times. May 9, 2023. https://www.nytimes.com/2023/05/09/climate/galapagos-ecuador-debt-nature.html.

[CR18] Elliott RJR, Schumacher I, Withagen C (2020). Suggestions for a Covid-19 post-pandemic research agenda in environmental economics. Environmental and Resource Economics.

[CR19] Essers D, Cassimon D, Prowse M (2021). Debt-for-climate swaps: killing two birds with one stone?. Global Environmental Change.

[CR21] Ferry M (2019). The carrot and stick approach to debt relief: overcoming moral hazard. Journal of African Economies.

[CR22] Fodha M, Seegmuller T (2014). Environmental quality, public debt and economic development. Environmental and Resource Economics.

[CR23] Freytag A, Pehnelt G (2009). Debt relief and governance quality in developing countries. World Development.

[CR20] F4B-Initiative. 2020. *Recapitalising sovereign debt—why nature performance bonds are needed now*. Finance for Biodiversity Initiative. https://a1be08a4-d8fb-4c22-9e4a-2b2f4cb7e41d.filesusr.com/ugd/643e85_e2f3eccae35c45a8b875a974a8918922.pdf

[CR24] Georgieva, K., M. Chamon, and V. Thakoor. 2022, December 14. *Swapping debt for climate or nature pledges can help fund resilience*. IMF. https://www.imf.org/en/Blogs/Articles/2022/12/14/swapping-debt-for-climate-or-nature-pledges-can-help-fund-resilience

[CR25] Global Forest Watch. n.d. *Democratic republic of the Congo deforestation rates & statistics*. https://www.globalforestwatch.org/dashboards/country/COD/. Accessed 18 May 2021

[CR26] Goldberg L, Lagomasino D, Thomas N, Fatoyinbo T (2020). Global declines in human-driven mangrove loss. Global Change Biology.

[CR27] Graham J (2022). Analysis: the next Amazon? Congo Basin faces rising deforestation threat. Reuters.

[CR28] Grill G, Lehner B, Lumsdon AE, MacDonald GK, Zarfl C, Liermann CR (2015). An index-based framework for assessing patterns and trends in river fragmentation and flow regulation by global dams at multiple scales. Environmental Research Letters.

[CR29] Hobi ML, Dubinin M, Graham CH, Coops NC, Clayton MK, Pidgeon AM, Radeloff VC (2017). A comparison of dynamic habitat indices derived from different MODIS products as predictors of avian species richness. Remote Sensing of Environment.

[CR30] Hughes AC (2017). Understanding the drivers of Southeast Asian biodiversity loss. Ecosphere.

[CR31] Hughes AC (2019). Understanding and minimizing environmental impacts of the belt and road initiative. Conservation Biology.

[CR74] Hughes, A.C. 2023. The post‐2020 global biodiversity framework: how did we get here, and where do we go next? 2020 年后全球生物多样性框架: 历史与展望. *Integrative Conservation* 2: 1–9. 10.1002/inc3.16

[CR32] Hughes AC, Orr MC, Yang Q, Qiao H (2021). Effectively and accurately mapping global biodiversity patterns for different regions and taxa. Global Ecology and Biogeography.

[CR33] Hughes AC, Orr MC, Ma K, Costello MJ, Waller J, Provoost P, Yang Q, Zhu C, Qiao H (2021). Sampling biases shape our view of the natural world. Ecography.

[CR34] IMF. (n.d.). *Debt sustainability analysis* [Text/HTML]. World Bank. https://www.worldbank.org/en/programs/debt-toolkit/dsa. Accessed 4 Nov 2020

[CR35] James A, Gaston KJ, Balmford A (2001). Can we afford to conserve biodiversity?. BioScience.

[CR36] Jenkins CN, Van Houtan KS, Pimm SL, Sexton JO (2015). US protected lands mismatch biodiversity priorities. Proceedings of the National Academy of Sciences.

[CR37] Jetz W (2002). Geographic range size and determinants of avian species richness. Science.

[CR38] Jetz W, McPherson JM, Guralnick RP (2012). Integrating biodiversity distribution knowledge: toward a global map of life. Trends in Ecology & Evolution.

[CR39] Jones, M., and R. Campos. 2023, May 9. Ecuador seals record debt-for-nature swap with Galapagos bond. *Reuters*. https://www.reuters.com/world/americas/ecuador-seals-record-debt-for-nature-swap-with-galapagos-bond-2023-05-09/

[CR40] Jung M, Arnell A, de Lamo X, García-Rangel S, Lewis M, Mark J, Merow C, Miles L, Ondo I, Pironon S, Ravilious C, Rivers M, Schepaschenko D, Tallowin O, van Soesbergen A, Govaerts R, Boyle BL, Enquist BJ, Feng X (2021). Areas of global importance for conserving terrestrial biodiversity, carbon and water. Nature Ecology & Evolution.

[CR41] Koffi, G. 2023. Scientists and policymakers join forces in the fight against DRC deforestation. Forest News. Friday, 12 May 2023. https://forestsnews.cifor.org/82280/scientists-and-policymakers-join-forces-in-fight-against-drc-deforestation?fnl

[CR42] Kukkala AS, Moilanen A (2013). Core concepts of spatial prioritisation in systematic conservation planning. Biological Reviews.

[CR43] Lagos RA (1992). Debt relief through debt conversion: a critical analysis of the Chilean debt conversion programme. The Journal of Development Studies.

[CR44] Landers, C., and N. Lee. 2021, November 10. *Belize’s big blue debt deal: at last, a scalable model?* Center For Global Development. https://www.cgdev.org/blog/belizes-big-blue-debt-deal-last-scalable-model

[CR45] Lazard. 2021. *Debt-for-SDGs swaps in indebted countries: the right instrument to meet the funding gap?* European Commission. https://europa.eu/capacity4dev/paramos/file/120008/download?token=U1eoc0ac

[CR46] Maxwell SL, Cazalis V, Dudley N, Hoffmann M, Rodrigues ASL, Stolton S, Visconti P, Woodley S, Kingston N, Lewis E, Maron M, Strassburg BBN, Wenger A, Jonas HD, Venter O, Watson JEM (2020). Area-based conservation in the twenty-first century. Nature.

[CR47] Nedopil, C., M. Yue, and T. Sun. 2023. *(Re)orienting sovereign debt to support the SDGs*. United Nations Development Programme (UNDP).

[CR48] Oldekop JA, Horner R, Hulme D, Adhikari R, Agarwal B, Alford M, Bakewell O, Banks N (2020). COVID-19 and the case for global development. World Development.

[CR49] Orr MC, Hughes AC (2022). Regreening: green is not always gold. Nature.

[CR50] Pressey RL, Visconti P, Ferraro PJ (2015). Making parks make a difference: poor alignment of policy, planning and management with protected-area impact, and ways forward. Philosophical Transactions of the Royal Society b: Biological Sciences.

[CR51] Richards DR, Thompson BS, Wijedasa L (2020). Quantifying net loss of global mangrove carbon stocks from 20 years of land cover change. Nature Communications.

[CR52] Shabecoff, P. 1987, July 14. Bolivia to protect lands in swap for lower debt. *The New York Times*, 2. https://www.nytimes.com/1987/07/14/science/bolivia-to-protect-lands-in-swap-for-lower-debt.html

[CR53] Shalal, A. 2021a, April 8. IMF, World Bank to unveil “green debt swaps” option by November, Georgieva says. *Reuters*. https://www.reuters.com/article/us-imf-world-bank-climate-swaps-idUSKBN2BV2NU

[CR54] Shalal, A. 2021b, October 29. IMF struggling over long-awaited “green debt swap” push as COP26 nears. *Reuters*. https://www.reuters.com/business/sustainable-business/imf-struggling-over-long-awaited-green-debt-swap-push-cop26-nears-2021b-10-29/

[CR55] Sheikh, P.A. 2018. *Debt-for-nature initiatives and the tropical forest conservation act: status and implementation* (p. 21). Washington: Congressional Research Service.

[CR56] Silveira EMO, Radeloff VC, Martinuzzi S, Martínez Pastur GJ, Rivera LO, Politi N, Lizarraga L, Farwell LS, Elsen PR, Pidgeon AM (2021). Spatio-temporal remotely sensed indices identify hotspots of biodiversity conservation concern. Remote Sensing of Environment.

[CR57] Simmons BA, Ray R, Yang H, Gallagher KP (2021). China can help solve the debt and environmental crises. Science.

[CR58] Smith R, Mistry J, Berardi A (2017). Assessing “overgrazing” in savannas. Savannas and dry forests: linking people with nature.

[CR59] Sommer JM, Restivo M, Shandra JM (2020). The United States, bilateral debt-for-nature swaps, and forest loss: a cross-national analysis. The Journal of Development Studies.

[CR60] Stubbs T, Kring W, Laskaridis C, Kentikelenis A, Gallagher K (2021). Whatever it takes? The global financial safety net, Covid-19, and developing countries. World Development.

[CR61] The Gecko Project. 2023. “Green” finance bankrolls forest destruction in Indonesia. Climate Home News. https://www.climatechangenews.com/2023/06/01/deforestation-indonesia-biomass-green-finance-papua-medco-indigenous/. Accessed 1 June 2023

[CR62] Thomas N, Lucas R, Bunting P, Hardy A, Rosenqvist A, Simard M (2017). Distribution and drivers of global mangrove forest change, 1996–2010. PLoS ONE.

[CR63] Transparency International. 2021. *2021 corruptions perceptions index—explore the results*. Transparency.Org. https://www.transparency.org/en/cpi/2021

[CR64] UNEP. 2023. Decades of mangrove forest change: what does it mean for nature, people and the climate? UNEP, Nairobi

[CR65] Venter O, Sanderson EW, Magrach A, Allan JR, Beher J, Jones KR, Possingham HP, Laurance WF, Wood P, Fekete BM, Levy MA, Watson JEM (2016). Global terrestrial Human Footprint maps for 1993 and 2009. Scientific Data.

[CR66] Volz, U., S. Akhtar, K.P. Gallagher, S. Griffith-Jones, and J. Haas. (2020). *Debt relief for green and inclusive recovery*. Heinrich Böll Foundation, the Center for Sustainable Finance at SOAS, University of London, Boston University Global Development Policy Center.

[CR67] Waldron A, Adams V, Allan J, Arnell A, Asner G, Atkinson S, Baccini A, Baillie J (2020). Protecting 30% of the planet for nature: costs, benefits and economic implications (Waldron Report 30 by 30).

[CR68] Watson JEM, Dudley N, Segan DB, Hockings M (2014). The performance and potential of protected areas. Nature.

[CR69] World Bank. 2020, December 21. *COVID 19: debt service suspension initiative* [Text/HTML]. World Bank. https://www.worldbank.org/en/topic/debt/brief/covid-19-debt-service-suspension-initiative

[CR70] World Bank. 2021, April 1. *Debt service suspension initiative* [Text/HTML]. World Bank. https://www.worldbank.org/en/topic/debt/brief/covid-19-debt-service-suspension-initiative

[CR71] World Bank. (n.d.). *Debt service payments projections: what do we measure*. World Bank. https://databank.worldbank.org/data/download/site-content/Debt%20Service%20Payments%20Projections-%20What%20do%20we%20measure.pdf

[CR72] Zhang Y, Tariq A, Hughes AC, Hong D, Wei F, Sun H (2023). Challenges and solutions to biodiversity conservation in arid lands. Science of the Total Environment.

[CR73] Zhu L, Hughes AC, Zhao X-Q, Zhou L-J, Ma K-P, Shen X-L, Li S, Liu M-Z, Xu W-B, Watson JEM (2021). Regional scalable priorities for national biodiversity and carbon conservation planning in Asia. Science Advances.

