# Supplementary material for: Are debt-for-nature swaps scalable: Which nature, how much debt, and who pays?
Source: Ambio. 2023 Sep 2;53(1):63–78. doi: 10.1007/s13280-023-01914-4 (PMC10692041; doi:10.1007/s13280-023-01914-4)
Supplement: Supplementary file 1 — Supplementary file1 (PDF 806 kb) [file 13280_2023_1914_MOESM1_ESM.pdf]

Supplements:

Are debt-for-nature swaps scalable: Which nature, how much debt, and who pays?

Authors:

1. Christoph Nedopil, 220 Handan Road, Yangpu District, Shanghai 200433, P.R. China,
2. Mengdi Yue, 220 Handan Road, Yangpu District, Shanghai 200433, P.R. China
3. Alice C. Hughes*, School of Biological Sciences, University of Hong Kong, Hong Kong. Achughes@hku.hk

This file includes:

Detailed Materials and Methods
Figure S1. Total public external debt-to-GDP ratios for 67 DSSI eligible countries by creditor type in 2019
Table S1. Amount of debt needed for DNS by individual creditors
Table S2. Cost of nature
Table S3. Assumed acquisition cost of protected areas in different regions
Supplementary References

Other Supplementary Materials for this manuscript include the following:

- Data S1. Priorities and debt on a national basis (Separate table)
- Data S2. Overlaps between species richness hotspots from IUCN/Birdlife and three indices of ecosystem productivity (Separate table)
- Data S3. Overlap between KBAs and priorities, and percentage forest loss since 2000 on a national basis (Separate table)
- Data S4. Debt to GDP of 67 countries eligible for debt for Nature swaps (Separate table)
- Data S5. Links to all data sources used in analysis (Separate table)
- Data S6. Public external debt data in 2019 for 67 DSSI eligible countries (Separate table)

Materials and Methods

Biodiversity prioritization analysis

Identifying priorities for conservation has been a topic of much discussion for over two decades (Margules *et al.,* 2000; Brooks et al., 2006). Global2000 and WWF analyses highlighted ecoregions of key importance (Gaston 2000) and since this point many alternate approaches have been used to generate hotspots. Zero-extinction alliance highlighted areas it deemed key to preventing extinction, whereas other prioritization indices highlighted hotspots of threat (Alliance for Zero Extinction 2018), as well as hotspots of diversity. The advent and growing popularity of species niche models combined with the growing availability of high resolution GIS data, and the growth of platforms such as GBIF for species distribution data provided the opportunity to model species distribution and richness at ever increasing resolutions (Heberling et al., 2021). Yet despite this, and the over 1 billion records in GBIF (Hughes et al., 2021), the vast majority of this data is from birds, and from developed countries. Thus, for larger scale analysis IUCN and birdlife data has become the mainstay of recent analyses, mapping priorities across regions based on species assumed to be present. Yet this data is not only not representative across taxa (Hughes et al., 2021b), but includes spatial biases which differ by region and taxa (Hughes et al., 2021c). Thus whilst many analyses have used such approaches, their ability to capture key areas for under-represented taxa, unassessed regions, and around political borders is questionable. Given the expediency for the need for conservation in some regions, and possible increases in economically driven land-conversion in some developing countries following the pandemic there is an urgent need to protect key areas before they are lost.

In identifying potential hotspots, we aimed to utilize the known relationship between diversity and productivity to identify undisturbed areas with high potential to host diversity. Such an

approach overcomes the biases in explicit biodiversity datasets, whilst identifying regions with the potential to maintain diversity, thus providing a more standard metric to develop priorities on global scales and between biomes. Protected areas are known to be able to prevent or reduce deforestation in areas at risk (Andam et al., 2008; Hughes 2018) and thus given the high rates of deforestation in many of these regions, and small degree of protected area coverage (Data S3) with countries listed showing up to 35% loss of forest cover since 2000 developing effective protected areas is essential.

Identifying hotspots for prioritization required two main steps (Silveira et al., 2021): firstly, to remove highly disturbed areas, and secondly to identify priority-areas within remaining intact habitats. To understand relative intactness we updated the Human Footprint map (Venter et al., 2016), using the same methods as was used in their footprint map with more recent layers (as previously published human footprint maps rely on layers over a decade old) and adding two extra informative layers (and removing rivers). To do this we first created a map of disturbance including population density (WorldPop, Soille et al., 2018), roads and other linear infrastructure (Meijer et al., 2018; World Food Program 2017), lights at night (NASA 2020), crops (Teluguntla et al 2015), pastureland (Venter et al., 2016), petrochemical areas (Lujala et al., 2007; Blossom et al., 2009), dams (Mulligan et al., 2020), and mines (Hughes 2019). all of which were used at a resolution of $1km^2$. Of these all maps except the pastureland map were updated to more recent versions relative to the Venter et al map (2016), as no more recent version of the pastureland existed.

A new updated human footprint was developed at a 1km resolution utilizing more recent datasets. Data were treated in a similar way to the original human footprint (Venter et al., 2016; Sanderson 2002), but with some slight changes to better reflect intensity in some layers, and the majority of layers were updated. We did not include waterways in the footprint, as these are natural features and development on them will be signaled by the lights at night layer as well as the impermeable surface (built up) areas layers, removing the need to artificially fragment large forest extents when they lack secondary development. But we retained the pasture map (Venter et al., 2016) for 2009 and replaced maps for roads (2019), railways (2019), built-up areas (2014), population (2020), cropland (2015) (Teluguntla et al 2015), and nights at light (2020) (NASA 2020). In addition to traditional variables used within the human-footprint, we included petrochemical areas (Lujala et al., 2007; Blossom et al., 2009) dams Mulligan et al 2020), and mines (Hughes 2019). Original links to data used are available within Data S5.

Similar to former human footprint analyses, each variable was given a value of up to ten based on the level of landscape modification. One layer, pastureland, was maintained from the 2009 analysis as no newer releases of comparable data were available. Each of these layers were scored with values of 0 (no modification) to 10 (intense modification), this was also based on the original human footprint methodology. The values within each layer were based on the original human footprint analysis where possible. In the pasture lands layer (using the same layer as the original human footprint dataset) with values of 2-4 were assigned to different categories of pastureland. For crops, we assigned irrigated crops a value of eight, rainfed a value of seven, and mosaics a value of six based on the scoring scheme from the original HFP. For the population, built-up area, and lights at night layers, each was classified into 10 different zones based on the values, with areas showing almost no modification always giving the value of 10. Equal intervals were used for classification for most factors, but the geometric interval was used for the human population as this was a better match for earlier versions of the human footprint, and better captured the important variations at lower populations (above higher thresholds, additional population will have negligible impact on biodiversity). Trying to

optimize this scaling is important not only to make it comparable to previous versions of the footprint, but also because as all variables naturally have different maximum and minimum values they must be scaled to enable comparability. However, thresholds that were too low would class relatively unimpacted areas as disturbed, and thus comparing the reclassified layers to old versions of the footprint and high-resolution imagery helps ensure these layers are representative.

For roads and railways, we used the line-density tool in ArcMap 10.3 to explore density at a 1km resolution and included a 5km buffer-area around each line to calculate density within the buffer. This was then reclassified into values between 6-8. Mines (Hughes 2019) and dams were also analysed in terms of density per square kilometer, so more intensely used areas have higher values.

The new human footprint map was then built by aggregating all the data layers together to form a single composite index of modification using the mosaic to new raster function. Areas with a value below 10 in this new composite map were considered as relatively intact and reclassified to zero, whereas all other values were reclassified to "NoData". Thus, this low-disturbance area provided a mask to assess priorities, as the degree of pressure on these systems were relatively low and likely to represent either no disturbance, or a single form of disturbance which may be possible to restore at a relatively low cost. This approach is also sensible, as it prioritizes areas with the greatest potential for restoration, and the lowest demand for their use (thus not preventing people from accessing resources they need), as well as low or no costs for restoration. This low-disturbance mask was used to extract three indices of productivity; NDVI (a surrogate of Net primary productivity (*Copernicus*)), Leaf area index (LAI) (Xu et al., 2018), and Enhanced vegetation index (EVI) (Wang et al., 2021) globally to identify highly productive natural ecosystems. These three indices were chosen because they are known correlates of biodiversity (Xu et al., 2018; Coops et al., 2018), but are less likely to be impacted by cloud cover than metrics live Fraction of photosynthetically active radiation (FPAR) (Xu et al., 2018; Coops et al., 2018). The 15 global biomes (Dinerstein et al., 2017) were used to calculate the average productivity for each biome using the zonal statistics function. These biomes were used to enable representation across ecosystems, whilst developing a framework simple enough to implement, as ecoregions would have created an intractable number of regions, and hinder comparison between regions. The 15 biomes were then separated into individual shapefiles, then used to produce a productivity layer for each biome by clipping the productivity for each biome. These productivity layers were then used to identify priority-areas for each biome relative to the mean productivity of each biome. These priority-areas were ranked into first and second rank priorities based on their productivity and to distinguish between the most productive and less productive areas within each biome.

Testing productivity as a surrogate of richness

For forest biomes, many areas had high productivity, so forest biomes were classified to show areas with 50-75% of maximum richness as the second tier, and 76-100% as 1st tier. For grassland and desert biomes, the mean NDVI of those biomes was divided in half, and the areas above average richness and top quartile of richness were used. For tundra and rock and ice biomes, the same threshold was used as forest (though very little of this biome fell within the 67 countries). These areas were then re-mosaiced to form a global priority-area layer for all 15 biomes combined. This was repeated for both other productivity layers. The leaf area index for 2020 was downloaded monthly via FTP (M0111222 GLOBE_PROBAV_V1.5.1 from the Copernicus site (Copernicus)), and average LAI calculated using the mosaic function in ArcMap 10.3. For EVI mean an EVI was reconstructed from the Coefficient of variation and

standard deviation files from (StDev/CoV) based on Tuanmu & Jetz 2015 (Tuanmu & Jetz., 2015) by first converting to each layer to float then using the divide function to obtain an EVI layer. Thus, all three productivity layers were at 1km prior to analysis. Whilst productivity is known to be an effective surrogate of biodiversity(Coops et al., 2018; Hobi et al., 2017; Silveira et al., 2021), finding the best index for diversity needs some caution. These three indices were then compared to diversity based on birdlife and IUCN data. Whilst these IUCN maps have inherent biases, finding what proportion fall within the priorities from each of the three productivity layers does help guide how well these areas may cover biodiversity hotspots. Biodiversity hotspots were mapped by stacking binary maps for Odonata (2,239 species), Reptilia (10,423 species), Amphibia (6,684 species), Mammalia (2239 species) and Aves (10,423 species) using codes and richness layers from Hughes et al., 2021. Richness was then clipped using the modified human footprint layer. The output of this was then treated in two ways for each taxa. Firstly, each was classified into four equal quartiles and the top two retained to form an "overall" hotspot for each group. In addition, each taxa was partitioned into the fourteen biomes, and the top two quartiles for each biome identified and classified to make a binary map for each taxa in each biome, before mosaicking to form biome specific hotspot maps for each taxa and represent the key areas of high diversity for each.

Categories from the three productivity layers were reclassified to values of 1,000 and 2,000 (for first and second tier priorities), then mosaicked to show when hotspots for each taxa overlapped with each of the productivity derived hotspot maps. The tabulate area tool was then used to calculate the areas of the various combinations of productivity hotspots and biodiversity hotpots in each country. The percentages of biodiversity hotspots falling inside the productivity hotspots for the three different types of productivity analysis for all biodiversity divisions were then calculated. This showed that NDVI covered the highest percentage of biodiversity hotspots, overall (with an average of 88.4%), and for both biome specific (92%) and global hotspots (84.8%), tables of comparisons are available in Data S2). This shows that whilst NDVI saturates at high levels, these areas are likely to be hyper-diverse, and thus high resolution above a certain threshold (as provided by EVI) actually limits the amount of diversity captured and makes comparison between biomes more challenging. Whilst NDVI only performed slightly better than EVI, its ability to better enable richness hotspots to be captured across biomes and taxa meant it was the most useful layer for further analysis to identify targets for potential DNS. NDVI had the greatest ability to capture diversity layers and is likely the best indicator for this resolution (Data S2).

To subdivide this into important functional classifications, we then masked the global priorities based on five different broad habitat types (forest, freshwater, mangrove, desert and grassland). This was done using high-resolution masks of each habitat, as global biome maps do not differentiate between potential landcover and areas which may have already been converted. To calculate forest and freshwater-riparian priorities, separate layers were used to mask the zones. For mangrove and freshwater-riparian systems, we combined two datasets to provide the most comprehensive coverage of such regions (Yamazaki et al., 2017; Gumbricht et al., 2017). The OSM water layer (Yamazaki et al., 2017) was reclassified to provide only lakes and rivers, then combined with the CIFOR river map to provide a separate classification for freshwater habitats and mangrove habitats and a mask generated for each of these different types of biome. For forests, a forest density layer was used (Crowther et al., 2015) and reclassified to show only forest areas (Hughes 2017) using a threshold density of 30,000 trees per ha. Then, recently deforested areas were removed to show only currently forested areas (Song et al., 2018) to provide a high-resolution global forest mask. These three habitat types (forest, freshwater and mangrove) as well as deserts and grasslands (based on Dinerstein et al., 2017 biome map, with the grassland biomes dissolved to form a single mask for grasslands)

were used to extract the priority-areas from the global zonal map. Higher-resolution datasets were used for forests and aquatic habitats because these can be challenging to map accurately at broad spatial-scales, and most low-disturbance grassland within the biome is likely to still represent high-value habitats, whereas the global biome map does not provide sufficient data for most aquatic biomes. Furthermore, forest degradation may be detectable from tree density or height, as well as differentiating natural forest from forestry plantations (which tree cover datasets such as global forest watch do not), but detecting over-grazing in grasslands would mainly be identifiable by changes in productivity, and thus already captured. Thus, in all cases we used the highest available resolution as a basis for generating the 1km masks to filter priorities for each intact biome.

This provided five habitat-specific layers (forest, desert, grassland, freshwater, and mangrove) as well as the zonal map. To identify priorities within this, we cross-referenced with protected areas by firstly dissolving all GIS protected areas listed within WDPA (UNEP-WCMC and IUCN 2021) then converting them to rasters and reclassifying all to a value of 100. This was then combined with the zonal priority map in addition to the five biome-specific maps using the sum function in mosaic to new raster so that protected priorities of each potential richness level and its protection status could be assayed.

The calculate area tool was then used to calculate the area of each zone of each priority within each country by overlaying each of the five "biome" priority maps, and the overall priority map with an administrative map of the world. This means that total priorities include biomes (such as tundra and rock and ice) which have priorities but were not analysed separately as they include little or no area in DSSI countries, and are also less diverse than other biomes. Furthermore, some biomes examined necessarily overlap: tall mangroves can be considered forests and host both aquatic and terrestrial species, and riparian areas include swamps, forests, and various other ecosystems which may therefore be listed as a priority under more than one dimension, and is likely to span greater diversity and heterogeneity.

To assess how well our priorities match previously identified key areas for biodiversity we assayed the overlap between our priorities and the Key Biodiversity Areas mapped through Birdlife International. KBAs were not used as a target as different countries have different approaches to KBA listing, and many KBAs evolved from important bird areas (IBAs), thus whilst the dataset is a useful comparator it is not representative at a taxonomic level (and many miss any assessments for many taxa), or at a national level when compared between countries. Relative to Key Biodiversity Areas (KBAs: http://www.keybiodiversityareas.org/), our priorities performed well in most countries, with a high degree of coverage in the most diverse biomes (Data S3). However, the overlap was notably lower in countries with dryer biomes. This is likely due to very small KBAs and ecosystems in some of these cases (i.e., single sections of a river), and a lower diversity than similar systems in other countries. Such systems are likely to rely on the identification of species based on intense surveys, and thus are not necessarily standardized between countries and regions. Furthermore, in these cases, such KBAs are often either already protected, or are at low risk due to inaccessibility, etc. Thus, in the regions with the highest levels of diversity and rates of habitat loss (i.e., deforestation for percentage forest loss since 2000, based on a data request from global forest watch (https://www.globalforestwatch.org/); see Data S3), our priorities perform well, and often include over 90% of existing KBAs, thus providing a complementary tool for the identification of priorities for protection, even without comprehensive and comparable data on species occurrence in many areas. This analysis shows that the priority-areas highlighted here perform well, and provide an effective way to map priorities at a large scale, without the need for direct biodiversity data. Furthermore, such an approach can be applied in concert with other remote

sensing approaches to enable coherent and standard metrics for the monitoring as well as prioritization of key areas for biodiversity at any scale.

Debt analysis

To understand "whose debt" is applicable for debt-for-nature swaps post COVID-19, we first identified "public external debt stock" as the type of debt relevant for analysis. In this term, "public" refers to debt owed by a public agency or a private agency with a public guarantee in the debtor country, and "external" refers to debt owed to nonresidents (Worldbank). By this, we make sure that our analysis aligns with the cases of past debt-for-nature swaps, which were generally implemented between the debtor government and one or more foreign lenders (usually the creditor governments).

We then narrowed down the group of debtor countries under analysis to the 73 countries eligible for Debt Service Suspension Initiative (DSSI) as they are more vulnerable to debt distress and have a higher priority for debt restructuring, including debt swaps. In response to the COVID-19, G20 established DSSI in May 2020 to provide temporary suspension of debt-service payments owed to official bilateral creditors until December 2021 (Worldbank 2020). In November 2020, the "Common Framework for Debt Treatments beyond the DSSI" was launched by G20 that also requires comparable debt relief from private creditors in addition to that from public creditors (IMF 2021).

Debt data for 67 out of the 73 DSSI eligible countries (detailed data for South Sudan, Micronesia, Tuvalu, Kiribati, and the Marshall Islands are not available and Kosovo is not universally recognized as a country) were obtained from the World Bank International Debt Statistics (IDS). IDS contains country-level data of public external debt stocks owed to four types of non-resident lenders: 1) official multilateral creditors, i.e., international organizations whose membership and decision-making process includes the government of two or more countries, such as the IMF, the World Bank, Asian Development Bank; 2) official bilateral creditors, i.e., lending by sovereign governments and all public institutions in which the government share is 50 percent or above and encompassed of the general government, central government; state and local government; central bank; and public enterprise; 3) bondholders, i.e., holdings of securities by investors for which the issuer has promised to pay a specified amount of money at a fixed date and income at periodic dates until maturity, including publicly placed bonds and privately placed bonds; and 4) non-official creditors include all other private creditors, including those that are officially supported by an export credit guarantee, or other forms of risk-mitigating guarantee, from an official bilateral entity or multilateral institution (Worldbank).

The sizes of total and disaggregated public external debts are measured as percentages of the debtor country's GDP (debt-to-GDP ratios), in line with the IMF Debt Sustainability Analysis (IMF). GDP data for individual debtor countries were obtained from the World Bank, except Somalia's from IMF. We used data in 2019 as they are the most current and complete data available while assuming that the overall debt levels and debt ratios in the 67 countries would be higher in 2020 due to economic recession and increased public spending.

The distribution of debt owed to different types of creditors in comparison to debtor country GDP is as follows (see Supplementary Figure 1).

1. **Official multilateral creditors**

Official multilateral creditors are multilateral bodies or banks, such as the World Bank or IMF, and account for 46% of total public external debt in the 67 DSSI countries. They are the largest creditors in 32 of the 67 countries.

Particularly, World Bank-IDA is the most important creditor in 21 countries, including Mozambique, Rwanda, Lesotho, Liberia and Malawi (top 5 with the highest level of IDA debt to GDP ratios). Inter-American Development Bank is the largest lender in Nicaragua, Guyana and Honduras; IMF is the largest creditor in Sierra Leone, Burundi, Kosovo and Central Africa Republic; Asian Development Bank is particularly prominent in Uzbekistan, Timor-Leste, Papua New Guinea and Solomon Islands.

2. Official bilateral creditors

Official bilateral creditors are state-level lenders and account for 34% of total public external debt in the 67 DSSI countries. Many emerging economies are borrowing money predominantly from other emerging markets. China, for example, is the major bilateral official lender in 20 countries including Djibouti, Republic of Congo, Lao PDR, Kyrgyz Republic and Tonga (top 5 with highest levels of Chinese debt-to-GDP ratios). India is the single largest creditor in Bhutan whose debt accounts for 74% of Bhutan's GDP in 2019. Russia is the largest creditor in Somalia and Afghanistan; Saudi Arabia is the largest creditor of Mauritania, while Haiti's debt is predominantly from Venezuela.

3. Bondholders

Private bondholders are individuals or companies based within a country and account for 13% of total public external debt in the 67 DSSI countries. They are the largest creditors in eight countries: Mongolia, Senegal, Zambia, St. Lucia, Ghana, Guinea-Bissau, Dominica, and Nigeria.

4. Non-official creditors

Non-official creditors account for only 7% of total public external debt in the 67 DSSI countries. They are the largest creditors in 2 countries: over a quarter of Cabo Verde's public external debt is owed to non-official lenders from Portugal; while the largest creditor in Chad is commercial banks in the UK.

To calculate how much debt each creditor would have to swap for nature, we assumed fair distribution of the swap across all creditors for every single country. Accordingly, we calculated the cost for each creditor in each debtor country and could sum up the total debt to be swapped under the different scenarios.

<u>Acquisition and management cost of protected areas</u>

To calculate the cost of nature conservation, we distinguished between the acquisition cost and management cost of protected areas, but we did not consider opportunity cost (Craigie et al., 2018; Waldron et al., 2020; Bohorquez et al., 2019).

To estimate these costs, we analyzed data from previous debt-for-nature swaps and nature conversion projects (Data S5). We further analyzed data for protected areas cost from numerous secondary sources, such as McCarthy et al.(2012), Gantioler et al. (2010), Frazee et al. (2003), and Waldron et al. (2020). In all cases, cost estimates varied widely, with a clear finding that data are incomplete and highly context-specific. For example, Waldron et al. find that to acquire 30% of nature (both water and land) by 2030 in over 100 emerging economies (including large countries, such as Brazil, Argentina and Chile, which are not part of our study),

prices range from USD300 to USD700 billion, acknowledging big differences between biomes and high costs particularly for marine ecosystem protection.

We further engaged in interviews with researchers and experts from nature conservation groups experienced in pricing nature conservation and previous application of DNS, such as WWF, Friends of Earth, The Nature Conservancy. We found that while prices are sometimes economically determined (e.g., based on alternate land use to conservation), the prices for DNS – and in particular for acquisition costs are often more politically determined for land that is not privately owned (the majority of our focus).

Accordingly, we expect prices to vary across countries and biomes. For desert protection, prices are likely lower given the land is less productive and under less pressure, while for more arable areas with high population growth prices might be slightly higher. Many of these pressures will be highest in the African continent, where a minority of potential agricultural land is currently used in some countries (i.e., D.R. Congo) and where demographically population is still increasing. However, the need to develop sustainably, especially to maintain key resources such as water, is an additional reason to use approaches such as debt-for-nature swaps to safeguard key ecosystem services and functions. Mineral resources etc. are not considered here, as it would be too speculative.

To further understand management costs, we directly reached out to operators of protected areas. We confirmed previous literature that operating costs depend on local personnel costs, vehicles (including boats for mangrove areas), and infrastructure costs to prevent unsustainable use through hunting or deforestation. Accordingly, operating costs will vary based on the frequency of patrols needed, the circumference of the area and the local salary expectations.

Accordingly, based on (a) interviews and desk research, (b) assumption of political willingness in debtor countries to engage in DNS, (c) the fact that the focus of our DNS is on conservation, not restoration (which should make it cheaper), and (d) that regional price differences would exist, we concluded that prices are, if anything, impossible to predict and that prices would be negotiable. To nevertheless provide relevant estimates for costs, we applied prices that allowed us to differentiate at least between regions as surveyed by James et al (2021) and Waldron et al.(2020). Accordingly, we adjusted their values to 2019 USD. To allow for a broader flexibility, we further calculated scenarios with prices 15% higher and 15% lower than those suggested by James et al. 2021

To estimate management cost, we followed the approach in Bohorquez et al. (2019) on ratios between acquisition and management cost of terrestrial protected areas. The authors found that depending on the survey, the ratio range from 0.49 to 25.5, with an average of 8.64. Conservatively, we estimate the average annual management cost for protected areas to be 31% of the acquisition cost.

To calculate regular service payments from the swapped debt (service payments are swapped from interest payments), we find in the World Bank data current interest rates in DSSI countries vary between 0 to 6%[1]. We take an average interest rate of 3%. We further assume a 30% discount on the debt-related payments, which is considerably more than on previous debt swaps: e.g., in the 2018 Seychelles DNS, the discount rate was 5.4%. This discount rate means that if USD1 million of debt would be swapped, the debtor country would have to pay 2.5% on USD0.7 million (USD17,500) per year to the environmental trust fund.

[1] For example, Angola pays USD1.372 million in 2020 for interest on a total public debt of USD36.4 million, equaling 4% interest payment: https://datatopics.worldbank.org/dssitables/annual/AGO, accessed May 18, 2021

Once all costs are established, the acquisition and management costs for protecting priority-areas for each country could be calculated, as well as the financing gap, particularly for the management costs.

Figure S1.
Total public external debt-to-GDP ratios for 67 DSSI eligible countries by creditor type in 2019 (data source: World Bank and IMF).

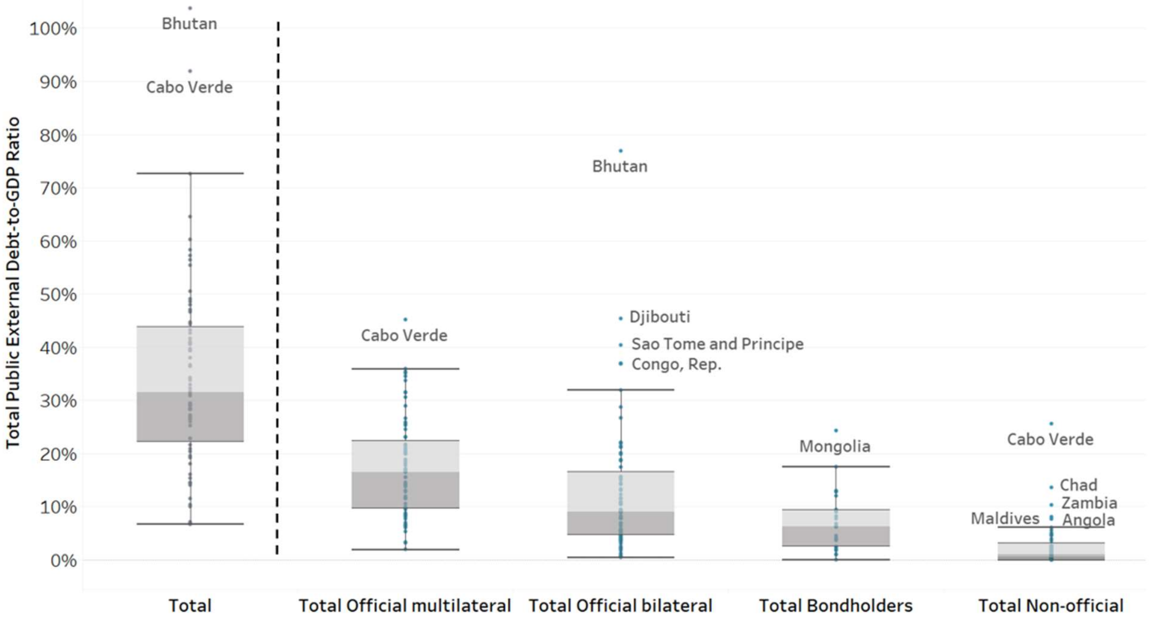

Table S1.
Amount of debt needed for DNS by individual creditors.
To calculate how much debt each creditor would have to swap for nature, we assumed fair distribution of the swap across all creditors for every single country. Accordingly, after filtering for a minimum of 2% applicable debt and maximize at 30% debt swap, we calculated the cost for each creditor in each debtor country and could sum up the total debt to be swapped under the different scenarios cost scenarios. We calculated both the debt needed for swapping under the assumption of no participation/participation of multilateral lenders.

		With participations of multilateral lenders			Without participation by multilateral lenders		
Type	Institution	baseline scenario	plus 15% scenario	minus 15 scenario	baseline scenario	plus 15% scenario	minus 15 scenario
Bondholders	Bondholders	926,344,007	1,042,794,348	832,182,559	1,573,570,092	1,768,531,119	1,418,926,197
Non-official	Angola	6,000,000	6,000,000	6,000,000	14,842,719	14,842,719	14,842,719
	Austria	5,647,626	6,494,770	4,347,723	9,119,827	10,487,802	7,148,678
	Bahamas	69,444	79,861	59,027	404,807	465,528	344,086
	Bahrain	-	-	-	-	-	-
	Barbados	-	-	-	-	-	-
	Belgium	14,477,134	16,699,637	12,305,564	33,190,027	38,471,548	28,211,523
	Brazil	233,240	268,226	198,254	420,004	483,005	357,004
	Canada	5,223,165	6,006,639	4,439,690	5,998,474	6,898,245	5,098,703
	China	132,297,666	150,306,879	79,336,066	251,217,011	284,918,035	153,380,414
	Cote D`Ivoire, Republic Of	4,056,206	4,664,637	3,447,775	10,728,432	12,337,697	9,119,167
	Czech Republic	23,741,100	23,741,100	23,741,100	97,751,411	97,751,411	97,751,411
	Denmark	8,888,032	10,221,237	7,554,827	29,288,751	33,682,063	24,895,438
	Egypt	1,759	2,022	-	3,194	3,673	-
	France	13,871,741	15,935,672	5,864,166	21,699,408	24,912,685	7,690,022
	Germany	16,293,560	18,938,447	13,849,526	59,546,306	69,673,183	50,614,360
	Greece	686,488	789,461	583,515	1,815,721	2,088,079	1,543,362
	Grenada	-	-	-	-	-	-
	Hong Kong	3,986,235	4,584,170	3,388,300	14,010,782	16,112,399	11,909,165
	Hungary	216,785	249,302	184,267	1,568,787	1,804,105	1,333,469
	India	312,367	357,287	57,636	869,831	994,215	364,344

	Ireland	14,168,867	16,294,197	12,043,537		41,164,768	47,339,483	34,990,052
	Israel	49,780,305	57,247,350	39,608,395		95,745,103	110,106,868	76,470,188
	Italy	19,268,081	22,158,294	9,412,424		38,936,693	44,777,197	20,444,066
	Japan	6,181,283	7,108,475	1,029,018		10,487,840	12,061,016	1,240,190
	Korea, Republic Of	2,036,717	2,342,225	1,731,210		2,610,190	3,001,718	2,218,661
	Liberia	32,726	37,635	27,817		236,824	272,347	201,300
	Mauritius	-	-	-		-	-	-
	Multiple lenders	89,832,220	99,998,473	79,665,967		550,069,830	618,965,920	481,173,740
	Netherlands	19,642,868	22,589,298	16,692,459		25,629,098	29,473,463	21,766,326
	Other Non-official	-	-	-		-	-	-
	Poland	227,918	262,106	193,730		1,649,356	1,896,760	1,401,953
	Portugal	21,746,573	25,008,559	18,484,587		30,071,752	34,582,515	25,560,989
	Saudi Arabia	79,654	91,603	67,706		116,542	134,023	99,060
	Serbia	15,579,600	16,186,914	14,226,966		34,196,435	35,250,817	31,552,296
	Singapore	151,616,135	151,858,555	151,373,715		619,679,957	619,990,635	619,369,279
	Slovenia	-	-	-		-	-	-
	South Africa	7,906,753	9,092,766	4,284,474		29,948,552	34,440,834	21,031,004
	Spain	29,988,458	34,486,726	25,490,189		34,473,401	39,644,411	29,302,391
	Sri Lanka	-	-	-		-	-	-
	St. Kitts And Nevis	-	-	-		-	-	-
	St. Lucia	-	-	-		-	-	-
	St. Vincent and The Grenadines	-	-	-		-	-	-
	Sweden	3,056,946	3,515,488	427,528		5,283,662	6,076,211	547,905
	Switzerland	23,658,729	27,120,463	4,205,601		42,841,513	49,052,335	7,583,723
	Thailand	1,023,493	1,177,017	869,969		1,311,675	1,508,427	1,114,924
	Trinidad and Tobago	3,761,700	3,761,700	3,761,700		11,840,093	11,840,093	11,840,093
	United Arab Emirates	392,797	451,716	118,452		680,552	782,634	187,168

	United Kingdom	353,233,860	406,917,647	294,251,659		602,704,016	699,699,768	502,352,554
	United States	55,687,337	63,885,188	22,140,403		94,069,653	107,691,446	34,403,467
Official bilateral	Argentina	1,354,500	1,354,500	1,354,500		4,263,340	4,263,340	4,263,340
	Australia	90,000,000	90,000,000	90,000,000		370,565,263	370,565,263	370,565,263
	Austria	8,309,612	9,632,165	6,561,964		11,249,908	13,390,205	8,542,421
	Belarus	-	-	-		-	-	-
	Belgium	2,141,955	2,463,248	1,742,148		10,826,924	12,450,963	8,839,579
	Brazil	32,091,445	36,905,161	27,277,728		54,218,434	62,351,199	46,085,669
	Canada	16,371,396	18,827,105	12,599,146		19,836,119	22,811,537	14,469,322
	China	2,644,796,283	3,009,835,830	2,209,482,003		5,228,580,426	6,033,107,995	4,446,624,578
	Czech Republic	8,023,142	8,718,951	6,818,559		13,516,588	14,442,828	11,487,080
	Denmark	-	-	-		-	-	-
	Egypt	1,414,745	1,429,799	1,202,534		3,068,941	3,101,597	2,608,600
	Finland	164,489	189,162	139,816		210,804	242,424	179,183
	France	180,717,402	207,188,729	146,609,201		385,028,944	453,051,266	306,241,753
	Germany	45,439,833	51,979,733	38,397,390		64,360,178	72,877,496	54,930,034
	Germany, Fed.Rep. Of	-	-	-		-	-	-
	Hungary	1,838,346	2,114,098	1,562,594		2,215,606	2,547,947	1,883,265
	India	201,162,939	226,963,123	137,592,403		515,249,203	581,281,932	391,182,392
	Iran, Islamic Republic Of	2,913,791	3,350,860	2,476,722		21,085,989	24,248,887	17,923,090
	Italy	4,948,024	5,575,433	1,821,661		9,234,140	10,257,939	3,671,208
	Japan	322,614,941	370,464,464	280,412,626		739,323,657	859,998,709	656,378,109
	Kuwait	53,874,462	59,128,573	45,014,576		166,734,739	187,217,962	138,059,132
	Libya	49,576,419	54,970,170	38,387,223		112,268,734	123,852,986	88,059,115
	Malaysia	1,061,190	1,220,368	902,011		1,278,963	1,470,808	1,087,119
	Multiple lenders	231,305,698	264,317,479	196,609,843		579,250,492	662,484,885	492,362,919

	Netherlands	636,888	732,421	541,355		799,215	919,097	679,332
	Nigeria	-	192,040	-		-	1,142,502	-
	Norway	2,853,048	3,281,005	2,425,091		4,838,390	5,564,148	4,112,631
	Other Bilateral	212,013,203	233,847,814	176,982,645		606,833,675	670,787,975	519,601,516
	Portugal	45,343,336	52,144,836	38,541,835		69,684,481	80,137,153	59,231,808
	Russian Federation	37,118,123	42,685,841	28,605,069		94,964,325	109,208,974	75,369,732
	Saudi Arabia	59,772,465	64,239,126	49,914,619		148,486,532	163,380,144	120,869,675
	Spain	3,812,141	4,383,962	3,240,320		7,306,300	8,402,245	6,210,355
	Sweden	1,099,417	1,264,330	934,505		7,054,995	8,113,244	5,996,745
	Switzerland	2,053,548	2,075,399	1,745,516		4,454,665	4,502,066	3,786,465
	Thailand	25,366,113	29,117,666	21,561,196		31,562,744	36,181,395	26,828,333
	Turkey	20,598,841	23,688,668	13,134,159		32,546,762	37,428,776	19,718,204
	United Arab Emirates	2,984,910	3,368,910	2,147,930		9,278,645	10,699,872	5,873,835
	United Kingdom	2,156,577	5,727,665	1,837,230		3,030,273	22,817,394	2,588,763
	United States	6,841,432	7,846,002	5,836,862		11,503,844	13,161,292	9,846,396
	Venezuela, RB	32,923,200	32,923,200	32,923,200		103,627,015	103,627,015	103,627,015
Official multilateral	African Dev, Bank	544,405,721	650,672,588	410,242,449				
	Asian Dev, Bank	510,551,820	523,843,173	528,769,376				
	International Monetary Fund	861,340,997	948,504,678	763,719,927				
	Other Multilaterals	514,486,736	581,610,200	420,520,015				
	World Bank-IBRD	77,206,515	87,976,682	81,874,513				
	World Bank-IDA	1,768,854,888	2,058,626,745	1,319,986,387				
	Inter-American Dev. Bank	163,854,000	163,854,000	163,854,000				

Table S2.

Cost of nature

Region	Country	Type	Time	Land Type	Land Use	Price in 2020 (USD per km^2)	Includes
America	Bolivia (*Shabecoff et al., 1987*)	DNS	1987	Tropical forest	Conservation areas for Buffer zones	130	Acquisition
Africa	Tanzania Rondo Plateau (*World Land trust 2021*)	Private Conservation group	2021 Fully funded	Coastal Forests	10 Reserves for lions, leopards, chameleons, birds and primate communities	2,220	Acquisition
South America	Bolivia (*ICFC*)	Private conservation group (ICFC)	2010-now Ongoing	Beni Savanna	Conservation of the blue-throated macaw	2,645	Acquisition and management
North America	Mexico (*Nature & Culture International*)	Commercial (NCI)	NA (ongoing)	Tropical dry forests land	Habitat for tropical rainforest	9,880	Acquisition and management
Asia	China (*MEE*)	government	1999-2003-	Cropland	Cropland conversion	11,115	Acquisition and conversion of agricultural land

Americas	Guatemala (*World Land Trust*)	Commercial (World Land Trust)	ongoing	Mangrove, Flooded Forest, Mountain Forest	Laguna Grande Sarstun Reserve	24,700	Acquisition, management, and fund management

Table S3.
Assumed acquisition cost of protected areas in different regions (*50*)

**Region**	**Acquisition cost per km^2 in USD**
Asia	2,293
CIS	899
Latin America/Caribbean	1,144
North Africa / Middle East	1,259
Pacific	17,395
Sub-Saharan Africa	1,535

Supplementary References

Alliance for Zero Extinction (2018) Global AZE map.https://zeroextinction.org/site-identification/2018-global-aze-map/

Andam, K. S., Ferraro, P. J., Pfaff, A., Sanchez-Azofeifa, G. A., & Robalino, J. A. (2008). Measuring the effectiveness of protected area networks in reducing deforestation. Proceedings of the national academy of sciences, 105(42), 16089-16094.

Bohorquez, J. J., Dvarskas, A., & Pikitch, E. K. (2019). Filling the data gap–a pressing need for advancing MPA sustainable finance. Frontiers in Marine Science, 45.

Brooks, T. M., Mittermeier, R. A., Da Fonseca, G. A., Gerlach, J., Hoffmann, M., Lamoreux, J. F., ... & Rodrigues, A. S. (2006). Global biodiversity conservation priorities. science, 313(5783), 58-61.

Coops, N. C., Kearney, S. P., Bolton, D. K., & Radeloff, V. C. (2018). Remotely-sensed productivity clusters capture global biodiversity patterns. Scientific reports, 8(1), 1-12.

Copernicus Global Land Operations “Vegetation and Energy” ”CGLOPS-1” Framework Service Contract N° 199494 (JRC).

Copernicus Global Land Service. Copernicus, (available at https://land.copernicus.vgt.vito.be/PDF/portal/Application.html#Home).

Craigie, I. D., & Pressey, R. L. (2018). Towards a better understanding of protected-area management costs (No. e26576v1). PeerJ Preprints.

Crowther, T. W., Glick, H. B., Covey, K. R., Bettigole, C., Maynard, D. S., Thomas, S. M., ... & Bradford, M. A. (2015). Mapping tree density at a global scale. Nature, 525(7568), 201-205.

Dinerstein, E., Olson, D., Joshi, A., Vynne, C., Burgess, N. D., Wikramanayake, E., ... & Saleem, M. (2017). An ecoregion-based approach to protecting half the terrestrial realm. BioScience, 67(6), 534-545.

Frazee, S. R., Cowling, R. M., Pressey, R. L., Turpie, J. K., & Lindenberg, N. (2003). Estimating the costs of conserving a biodiversity hotspot: a case-study of the Cape Floristic Region, South Africa. Biological Conservation, 112(1-2), 275-290.

Gantioler, S., Rayment, M., ten Brink, P., McConville, A., Kettunen, M., & Bassi, S. (2014). The costs and socio-economic benefits associated with the Natura 2000 network. International Journal of Sustainable Society, 6(1-2), 135-157.

Gaston, K. J. (2000). Global patterns in biodiversity. Nature, 405(6783), 220-227.

Green, M. J. B., James, A. N., & Paine, J. (1999). A Global Review of Protected Area Budgets and Staff. WCMC Biodiversity Series 10.

Gumbricht, T., Román-Cuesta, R. M., Verchot, L. V., Herold, M., Wittmann, F., Householder, E., ... & Murdiyarso, D. (2017). Tropical and subtropical wetlands distribution version 2.

Heberling, J. M., Miller, J. T., Noesgaard, D., Weingart, S. B., & Schigel, D. (2021). Data integration enables global biodiversity synthesis. Proceedings of the National Academy of Sciences, 118(6), e2018093118.

Hobi, M. L., Dubinin, M., Graham, C. H., Coops, N. C., Clayton, M. K., Pidgeon, A. M., & Radeloff, V. C. (2017). A comparison of Dynamic Habitat Indices derived from different MODIS products as predictors of avian species richness. Remote Sensing of Environment, 195, 142-152.

Hughes, A. C. (2017). Understanding the drivers of S outheast A sian biodiversity loss. Ecosphere, 8(1), e01624.

Hughes, A. C. (2018). Have Indo-Malaysian forests reached the end of the road?. Biological Conservation, 223, 129-137.

Hughes, A. C. (2019). Understanding and minimizing environmental impacts of the Belt and Road Initiative. Conservation Biology, 33(4), 883-894.

Hughes, A. C., Orr, M. C., Ma, K., Costello, M. J., Waller, J., Provoost, P., ... & Qiao, H. (2021). Sampling biases shape our view of the natural world. Ecography, 44(9), 1259-1269.

Hughes, A. C., Orr, M. C., Yang, Q., & Qiao, H. (2021c). Effectively and accurately mapping global biodiversity patterns for different regions and taxa. Global Ecology and Biogeography, 30(7), 1375-1388.

Hughes, A. C., Qiao, H., & Orr, M. C. (2021b). Extinction targets are not SMART (Specific, measurable, ambitious, realistic, and time Bound). BioScience, 71(2), 115-118.

ICFC, Bolivia: Conserving the Blue-throated Macaw and the Beni Savanna. International Conservation Fund of Canada (n.d.), (available at https://icfcanada.org/our-projects/projects/barba_azul).

IMF, (2021) Questions and Answers on Sovereign Debt Issues. IMF (available at https://www.imf.org/en/About/FAQ/sovereign-debt).

IMF, Debt Sustainability Analysis. World Bank, (available at https://www.worldbank.org/en/programs/debt-toolkit/dsa).

J. Blossom, Global Oil Pipelines. WorldMap Harvard (2009), (available at http://worldmap.harvard.edu/data/geonode:global_oil_pipelines_7z9).

Lujala, P., R∅ d, J. K., & Thieme, N. (2007). Fighting over oil: Introducing a new dataset. Conflict Management and Peace Science, 24(3), 239-256.

Margules, C. R., & Pressey, R. L. (2000). Systematic conservation planning. Nature, 405(6783), 243-253.

McCarthy, D. P., Donald, P. F., Scharlemann, J. P., Buchanan, G. M., Balmford, A., Green, J. M., ... & Butchart, S. H. (2012). Financial costs of meeting global biodiversity conservation targets: current spending and unmet needs. Science, 338(6109), 946-949.

MEE. China Ministry of Foreign Affairs, Building a Shared Future for All Life on Earth: China in Action (20AD), (available at https://www.fmprc.gov.cn/mfa_eng/wjdt_665385/2649_665393/t1816598.shtml).

Meijer, J. R., Huijbregts, M. A., Schotten, K. C., & Schipper, A. M. (2018). Global patterns of current and future road infrastructure. Environmental Research Letters, 13(6), 064006.

Mulligan, M.; van Soesbergen, A.; Sáenz, L. (2020): GOODD, a global dataset of more than 38,000 georeferenced dams. figshare. Collection. https://doi.org/10.6084/m9.figshare.c.4648214.v1

NASA EarthData, Night Lights 2020 Map. LAADS DAAC (2020), (available at https://ladsweb.modaps.eosdis.nasa.gov/missions-and-measurements/science-domain/nighttime-lights/).

Nature & Culture International, Conservation of Endangered Forests in Sierra de Alamos, Mexico. Nature & Culture International (n.d.), (available at http://www.naturalezaycultura.org/concept/htm/mexico/mexico.htm).

Sanderson, E. W., Redford, K. H., Vedder, A., Coppolillo, P. B., & Ward, S. E. (2002). A conceptual model for conservation planning based on landscape species requirements. Landscape and urban planning, 58(1), 41-56.

Shabecoff, P. (1987). Bolivia to Protect Lands in Swap for Lower Debt. The New York Times, 14.

Silveira, E. M., Radeloff, V. C., Martinuzzi, S., Pastur, G. J. M., Rivera, L. O., Politi, N., ... & Pidgeon, A. M. (2021). Spatio-temporal remotely sensed indices identify hotspots of biodiversity conservation concern. Remote Sensing of Environment, 258, 112368.

Soille, P., Burger, A., De Marchi, D., Kempeneers, P., Rodriguez, D., Syrris, V., & Vasilev, V. (2018). A versatile data-intensive computing platform for information retrieval from big geospatial data. Future Generation Computer Systems, 81, 30-40.

Song, X. P., Hansen, M. C., Stehman, S. V., Potapov, P. V., Tyukavina, A., Vermote, E. F., & Townshend, J. R. (2018). Global land change from 1982 to 2016. Nature, 560(7720), 639-643.

Tuanmu, M. N., & Jetz, W. (2015). A global, remote sensing‐based characterization of terrestrial habitat heterogeneity for biodiversity and ecosystem modelling. Global Ecology and Biogeography, 24(11), 1329-1339.

UNEP-WCMC and IUCN, Protected Planet: The World Database on Protected Areas (WDPA) and World Database on Other Effective Area-based Conservation Measures (WD-OECM)) [Online] (2021), (available at www.protectedplanet.net).

Venter, O., Sanderson, E. W., Magrach, A., Allan, J. R., Beher, J., Jones, K. R., ... & Watson, J. E. (2016). Global terrestrial Human Footprint maps for 1993 and 2009. Scientific data, 3(1), 1-10.

Waldron, A., Adams, V., Allan, J., Arnell, A et.al., (2020) Protecting 30% of the planet for nature: costs, benefits and economic implications (Waldron Report 30 by 30)" (Cambridge University, Cambridge), (available at https://www.conservation.cam.ac.uk/files/waldron_report_30_by_30_publish.pdf).

Wang, Y., Li, R., Hu, J., Wang, X., Kabeja, C., Min, Q., & Wang, Y. (2021). Evaluations of MODIS and microwave based satellite evapotranspiration products under varied cloud conditions over East Asia forests. Remote Sensing of Environment, 264, 112606.

World Bank, Debt Service Payments Projections: What do we measure , (available at https://databank.worldbank.org/data/download/site-content/Debt%20Service%20Payments%20Projections-%20What%20do%20we%20measure.pdf).

World Bank,(2020), COVID 19: Debt Service Suspension Initiative. World Bank (available at https://www.worldbank.org/en/topic/debt/brief/covid-19-debt-service-suspension-initiative).

World Food Programme (WFP), Global Railways (2017), (available at https://geonode.wfp.org/layers/geonode:wld_trs_railways_wfp/fp#more).

World Land Trust, Laguna Grande Sarstún Reserve. World Land Trust (n.d.), (available at https://www.worldlandtrust.org/tabs/laguna-grande-sarstun/).

World Land Trust, Tanzania's Coastal Forests: 49,000+ acres saved as WLT supporters help exceed appeal target within weeks. World Land Trust (2021), (available at https://www.worldlandtrust.org/news/2021/05/tanzanias-coastal-forests-appeal-target-exceeded/).

WorldPop, Population Density. (available at https://www.worldpop.org/project/categories?id=18).

Xu, B., Park, T., Yan, K., Chen, C., Zeng, Y., Song, W., ... & Myneni, R. B. (2018). Analysis of global LAI/FPAR products from VIIRS and MODIS sensors for spatio-temporal consistency and uncertainty from 2012–2016. Forests, 9(2), 73.

Yamazaki, D., Ikeshima, D., Tawatari, R., Yamaguchi, T., O'Loughlin, F., Neal, J. C., ... & Bates, P. D. (2017). A high‐accuracy map of global terrain elevations. Geophysical Research Letters, 44(11), 5844-5853.
